# Selective Targeting of Cancer Cells by Oxidative Vulnerabilities with Novel Curcumin Analogs

**DOI:** 10.1038/s41598-017-01230-4

**Published:** 2017-04-24

**Authors:** Christopher Pignanelli, Dennis Ma, Megan Noel, Jesse Ropat, Fadi Mansour, Colin Curran, Simon Pupulin, Kristen Larocque, Jianzhang Wu, Guang Liang, Yi Wang, Siyaram Pandey

**Affiliations:** 1 Department of Chemistry and Biochemistry, University of Windsor, 401 Sunset Avenue, Windsor, Ontario N9B 3P4 Canada; 2 Chemical Biology Research Center, School of Pharmaceutical Sciences, Wenzhou Medical University, University Town, Chashan, Wenzhou, Zhejiang 325035 P.R. China

## Abstract

Recently, research has focused on targeting the oxidative and metabolic vulnerabilities in cancer cells. Natural compounds like curcumin that target such susceptibilities have failed further clinical advancements due to the poor stability and bioavailability as well as the need of high effective doses. We have synthesized and evaluated the anti-cancer activity of several monocarbonyl analogs of curcumin. Interestingly, two novel analogs (Compound A and I) in comparison to curcumin, have increased chemical stability and have greater anti-cancer activity in a variety of human cancer cells, including triple-negative, inflammatory breast cancer cells. In particular, the generation of reactive oxygen species was selective to cancer cells and occurred upstream of mitochondrial collapse and execution of apoptosis. Furthermore, Compound A in combination with another cancer-selective/pro-oxidant, piperlongumine, caused an enhanced anti-cancer effect. Most importantly, Compound A was well tolerated by mice and was effective in inhibiting the growth of human triple-negative breast cancer and leukemia xenografts *in vivo* when administered intraperitoneally. Thus, exploiting oxidative vulnerabilities in cancer cells could be a selective and efficacious means to eradicate malignant cells as demonstrated by the curcumin analogs presented in this report with high therapeutic potential.

## Introduction

Recently, much work has been done exemplifying metabolic and oxidative stress vulnerabilities as promising targets for triggering physiological cell death in cancers selectively^1^. Oxidative stress can be a result of over production of reactive oxygen species (ROS) and/or a decrease in antioxidant defense systems. When the redox systems are imbalanced, this can lead to damage of macromolecules that can negatively influence whole organisms^2^. Cellular ROS can be synthesized intrinsically through various organelles including but not limited to the mitochondria, endoplasmic reticulum and peroxisomes or as a result of environmental factors including UV radiation, tobacco, xenobiotics, metals and ions^3, 4^. Interest in exploiting ROS to target cancer cells selectively has risen since cancer cells often exhibit higher levels of oxidative stress^5^. Consequently, this could render cancer cells more vulnerable to exogenous sources of ROS or stimuli that promote oxidative stress^5^. The combination of agents that increase ROS and those that suppress antioxidant defenses has been shown to be an effective treatment of different blood cancers with limited effects on normal lymphocytes^6–8^. These results have led to further successful clinical trials in lung, breast, and pancreatic cancers^9^.

The use of nutraceutical or derived compounds alone or in combination with chemotherapies has been used pre-clinically, clinically and anecdotally for years with encouraging results^10^. The natural compound, curcumin, isolated from the *Curcuma longa* plant is one molecule that has been significantly studied. Curcumin has been shown to directly or indirectly modify the activity of a variety of signaling molecules, including but not limited to transcription factors, enzymes, cell cycle regulatory proteins, cell survival proteins, and inflammatory molecules yielding various pleiotropic downstream effects^11^. Curcumin has been previously characterized to have antioxidant effects, although there have been reports of potential pro-oxidant properties^12–15^. Despite the extensive and promising pre-clinical findings of curcumin, it performed very poorly in clinical trials as a mono or combinatorial therapy as a result of its poor bioavailability and stability leading to serum concentrations below its critical pharmacological concentration^16–18^. Efforts have been made to increase curcumin’s bioavailability through the use of nanoparticles, liposomes, micelles, and phospholipid complexes. Increasing the amount and length of curcumin in circulation can lead to a subsequent increase in tissue distribution and biological activity^19^. Despite promising results^20–23^, it still remains to be seen whether these formulations will potentiate the clinical application of curcumin as a chemotherapeutic. Another strategy employed to potentially overcome these bottlenecks is the synthesis of curcumin analogs.

We have synthesized and screened several different analogs of curcumin. Here we report for the first time two novel analogs of curcumin that display increased chemical stability and enhanced cancer specific apoptosis inducing activity in comparison to curcumin. Notably, these analogs were capable of efficiently killing triple-negative, inflammatory breast, p53-negative colorectal, and various blood cancer cell lines. Interestingly, these analogs induce apoptosis primarily by increasing ROS selectively in cancerous cells. Furthermore, these results are complimented by the gene expression analysis that indicated Compound A induced differential expression of important genes related to redox systems specifically in cancer cells. Additionally, when Compound A is combined with piperlongumine, another pro-oxidant molecule there was a significant enhancement in cell death, selectively in cancer cells. Most importantly, Compound A administered intraperitoneally was able to effectively decrease xenograft human breast and leukemia cell tumor growth in mice. This observation potentially indicates that Compound A may be absorbed and stable within physiological systems. Furthermore, there were no observable changes in weight gain and behaviour, indicating overall tolerability. Therefore, these novel analogs of curcumin represent a class of compounds with potent anti-cancer activity through ROS generation in cancer cells.

## Results

### Synthesis of Curcumin Analogs Through a Substitution of β-diketone Moiety and Side Chain Substituents

**Figure 1:**
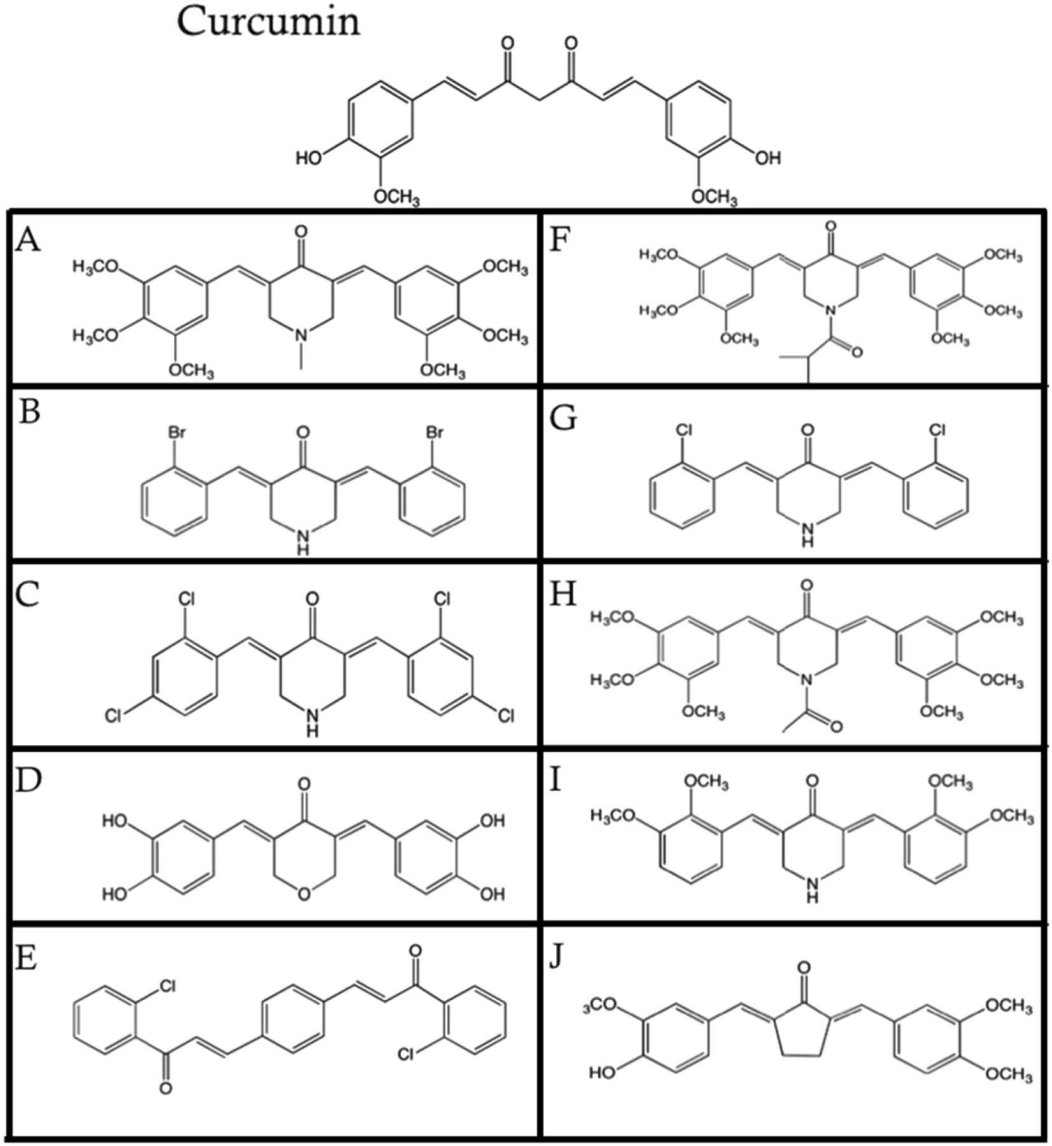
Chemical Structure of Curcumin and Analogs of Curcumin.

Monocarbonyl analogs of curcumin (MACs) have emerged in the past decade with a signature structure through the elimination of the β-diketone and replaced with a monocarbonyl group, along with varying substitutions on each aromatic ring, some of which have shown to have similar anti-cancer effects in comparison to natural curcumin^24, 25^. We synthesized several possible MACs in hope of obtaining more potent compounds (Fig. 1). Of these, Compounds A-E, G and I were found to be novel compounds and were further studied. Each analog varies in structure with several containing heterocyclic monocarbonyl rings including Compound A, B, C, D, F, G, H and I as well as varying aromatic substitutions including, hydroxyl, methoxy, or halogen groups.

### Curcumin Analogs Show More Potent Cytotoxicity Against Numerous Cancer Cell Lines Than Natural Curcumin

**Figure 2:**
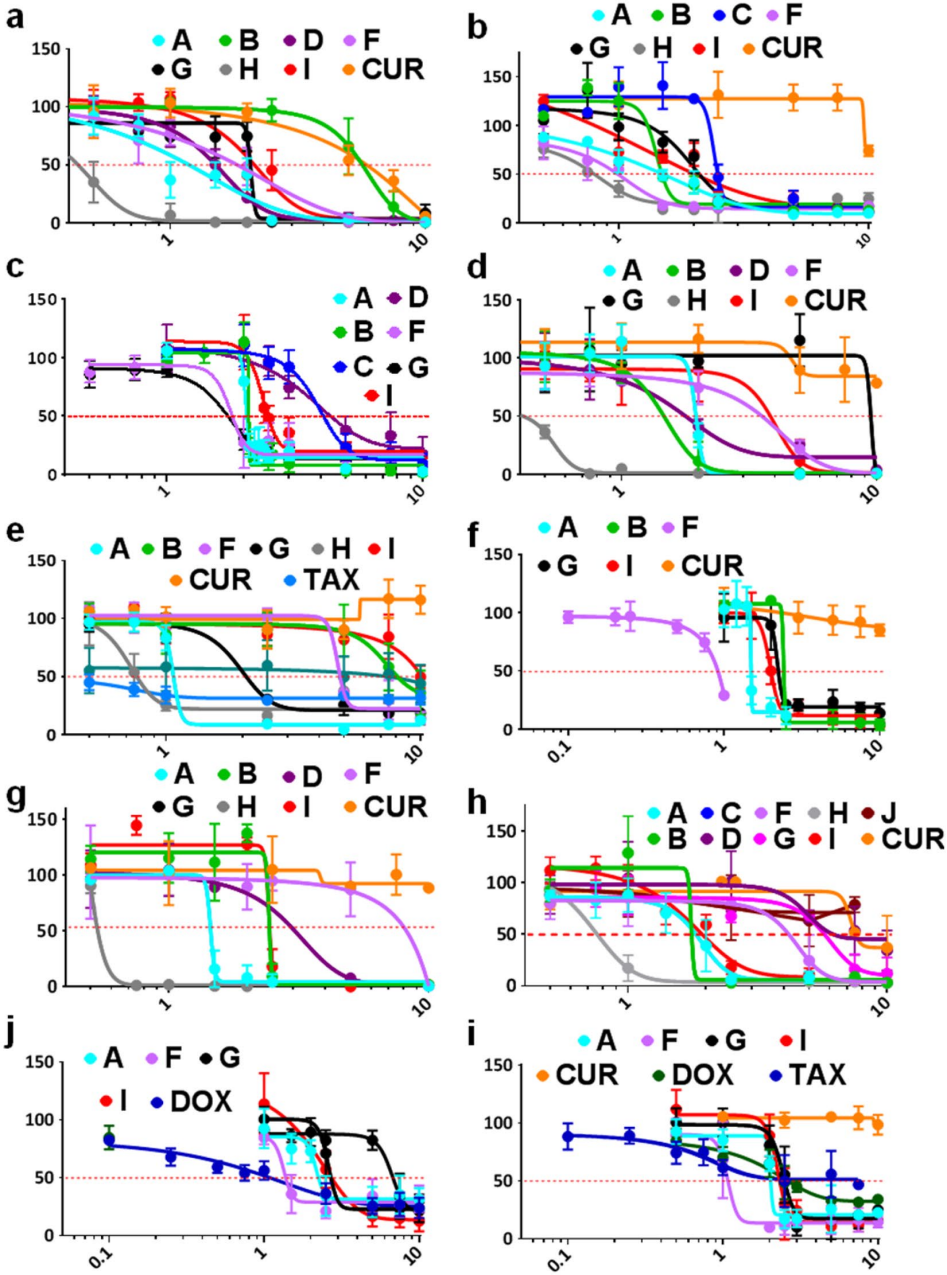
Curcumin Analogs Display Broad Efficacy in Reducing Cell Viability in 10 Cancerous Cell Lines Compared to Natural Curcumin. Cells were treated with various doses of curcumin, curcumin analogs, or common chemotherapeutics for 48 hours. Following treatment, WST-1 cell proliferation assay was used and the absorbance at 450 nm was measured. Y-axis is the percent mean ± SD from three independent experiments to the DMSO control and the x-axis is the concentrations used in micromolar. Results graphed using the log(inhibitor) vs response – Variable slope (four parameters) curve using GraphPrism6. (**a**) MV-4-11 (**b**) NCI-H23 (**c**) SAOS-2 (**d**) E6-1 (**e**) A549 (**f**) MG-63 (**g**) U-937 (**h**) HCT-116 (**i**) MDA-MB-231 (**j**) MDA-MB-468.

To evaluate the cytotoxicity of these analogs of curcumin and curcumin, the water-soluble tetrazolium-1 (WST-1) assay was utilized. Several analogs demonstrated a greater potency in decreasing cell viability across a panel of cancer cell lines including different blood (Fig. 2a,d,g), estrogen, progesterone, and HER-2 receptor negative (triple-negative) breast (Fig. 2i,j), bone (Fig. 2c,f), lung (Fig. 2b,e), and colon (Fig. 2h) cancer cell lines. In brief, of the 10 cancerous cell lines tested, the half maximal inhibitory concentration (IC_50_) of natural curcumin was found to be greater than 10 μM in all with the exception to the MV-4–11 leukemia cell line. Several of the curcumin analogs tested were found to have IC_50_ values significantly less than 10 μM. Interestingly, the IC_50_ for Compound A was found to be approximately 1–2 μM across each cancer cell lines tested. Similarly, Compound I also demonstrated greater anti-cancer activity than natural curcumin but less potent than Compound A. Additionally, Compound H had very potent anti-cancer activity (IC_50_ < 1 μM), however, it was found to be equally toxic to normal cells (*data not shown*). Therefore, Compounds A and I were chosen for further characterization and detailed study as presented in the following sections. A summary of all IC_50_ values can be found in Table 1.

**Table 1:** Summary of the half-maximal inhibitory concentration (IC_50_) at 48 hours.

IC_50_ (µM)	A549	NCI-H23	MV-4-11	E6-1	U-937	MG-63	SAOS-2	MBA-MD-231	MBA-MD-468	HCT-116
A	1.78 (±0.56)	1.49 (±0.15)	1.20 (±0.06)	1.94 (±0.034)	1.42 (±0.03)	1.49 (±0.01)	2.09 (±0.05)	1.82 (±0.27)	2.16 (±0.53)	1.65 (±0.43)
B	8.05 (±0.56)	1.50 (±0.014)	5.54 (±1.01)	1.30 (±0.32)	2.45 (±0.02)	2.32 (±0.08)	2.09 (±0.001)	>10	>10	1.42 (±0.32)
C	>10	2.51 (±0.05)	>10	>10	>10	Not Tested	4.14 (±1.01)	>10	Not Tested	Not Tested
D	>10	>10	1.45 (±0.18)	1.84 (±0.12)	1.63 (±0.10)	Not Tested	4.41 (±1.01)	>10	>10	8.11 (±2.81)
E	>10	Not Tested	>10	>10	>10	>10	>10	>10	>10	Not Tested
F	4.40 (±0.47)	0.88 (±0.19)	1.8 (±0.41)	3.45 (±1.05)	7.46 (±2.88)	0.83 (±0.4)	1.78 (±0.48)	0.99 (±0.32)	2.96 (±0.67)	5.40 (±1.02)
G	1.88 (±0.39)	2.09 (±0.18)	1.97 (±0.15)	9.46 (±0.14)	>10	2.40 (±0.39)	1.64 (±0.25)	2.39 (±0.18)	3.88 (±0.74)	5.19 (±0.06)
H	0.80 (±0.053)	0.76 (±0.001)	0.44 (±0.1)	0.39 (±0.14)	0.51 (±0.03)	Not Tested	Not Tested	Not Tested	Not Tested	0.65 (±0.20)
I	10.03 (±0.92)	2.21 (±0.16)	2.1 (±0.32)	3.09 (±1.24)	2.26 (±0.14)	2.28 (±0.47)	2.35 (±0.19)	2.57 (±0.31)	3.40 (±0.89)	2.24 (±0.48)
J	>10	>10	>10	>10	>10	>10	>10	>10	>10	>10
CUR	>10	>10	6.11 (±0.63)	>10	>10	>10	>10	>10	>10	>10
TAX	0.61 (±0.17)	Not Tested	Not Tested	Not Tested	Not Tested	Not Tested	Not Tested	0.64 (±0.16)	1.15 (±0.19)	Not Tested
DOX	Not Tested	Not Tested	Not Tested	Not Tested	Not Tested	Not Tested	Not Tested	2.03 (±0.65)	1.13 (±0.023)	Not Tested

### Compounds A and I Induce Apoptosis Selectively in Cancerous Cells

**Figure 3:**
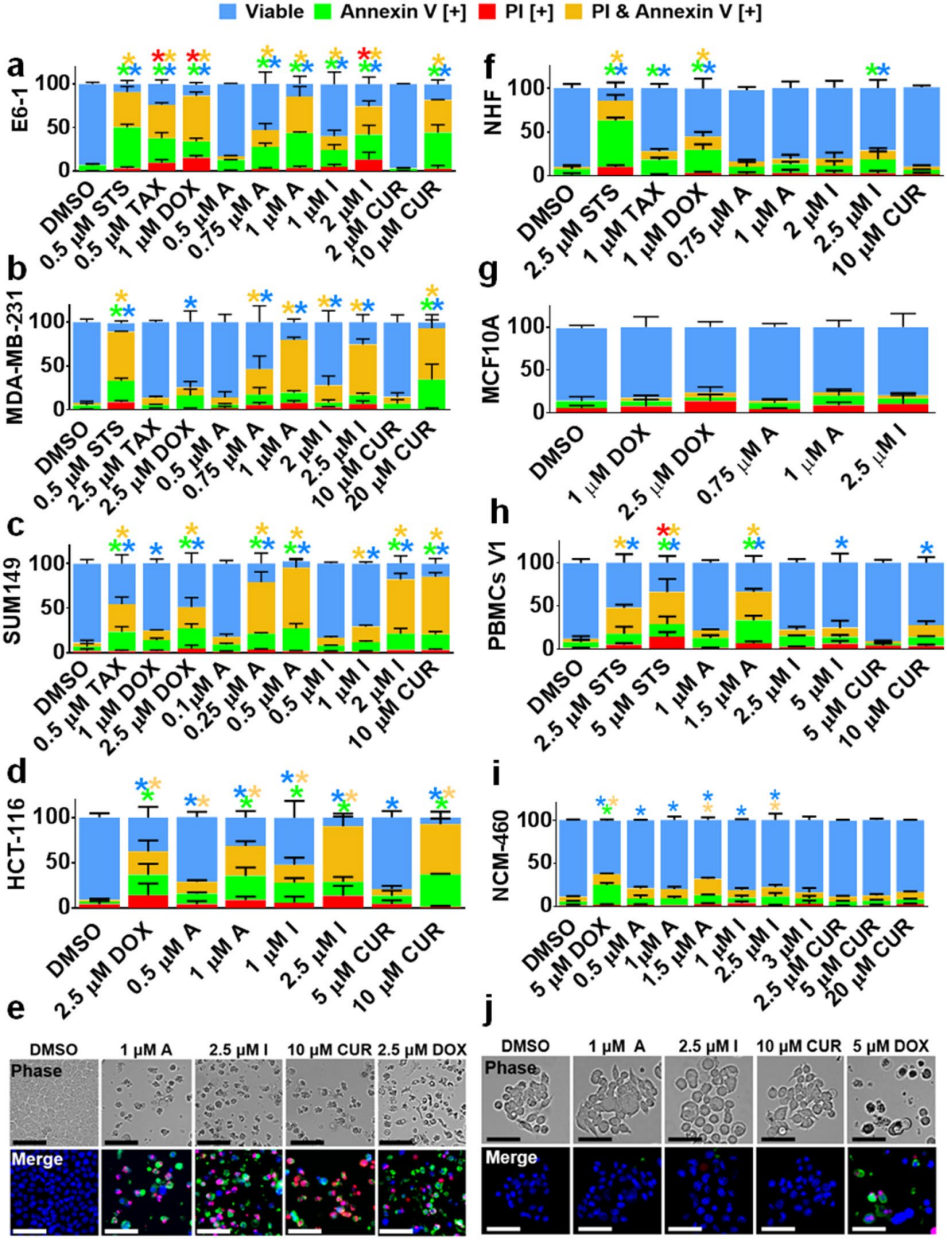
Curcumin Analogs Induce Apoptosis Selectively in Several Cancer Cell Lines. Following treatment with specified doses, cells were stained for Annexin V and PI. (**a–d**) Cancerous cell lines tested. Results were obtained using image-based cytometry. (**e**) HCT-116 micrographs at 72 hours. Top: Bright field images at 400x magnification. Bottom: Fluorescent images stained with Annexin V (green), PI (red), and Hoechst (blue) at 400x magnification. (**f–i**) Non-cancerous cell lines tested. Results were obtained using image-based cytometry. (**j**) Normal colon cells (NCM460) micrographs at 72 hours. Top: Bright field images at 400x magnification. Bottom: Fluorescent images stained with Annexin V (green), PI (red), and Hoechst (blue) at 400x magnification. For each graph the Y-axis is representative of percent of cells positive for Annexin V (green), PI (red), Annexin V and PI (yellow), or negative for both Annexin V and PI (blue). Values are expressed as a mean ± SD from three independent experiments. Results shown are at 48 hours with the exception to HCT-116 and NCM460, which were at 72 hours. For the microscopic images the scale bar is 50 microns. Images are representative of three independent experiments. Statistical calculations were performed using Two-Way ANOVA multiple comparison. *p < 0.05 vs Control (DMSO).

To determine the mode of cell death induced by Compounds A and I we carried out analysis of biochemical and morphological apoptosis and necrosis markers. Cells were stained with Annexin V and PI and subjected to image-based cytometry and microscopy following the treatment with various compounds (Fig. 3). Compounds A and I were able to cause significant induction of apoptotic markers at much lower doses (0.25–2.5 μM) than natural curcumin which produced these markers only at ≥10 μM (Fig. 3a–e). Most importantly, at these effective doses, Compound A and I induced very limited apoptosis in different non-cancerous cells similar to the solvent control (fibroblast, breast, and colon cells, as well as PBMCs isolated from a healthy volunteer, Fig. 3f–i). Interestingly, Compounds A and I showed greater apoptosis inducing activity in E6-1, MDA-MB-231 and MDA-MB-468 cell lines than the respective standard chemotherapeutics including doxorubicin (DOX) and taxol (TAX). Furthermore, in the triple-negative, inflammatory breast cancer cell line, SUM149, Compound A was approximately ten-fold more efficacious in inducing apoptosis compared to DOX (Fig. 3c). These quantitative results were supported by morphological changes shown by bright field and fluorescent microscopy in Fig. 3e,j, which revealed cell shrinkage and Annexin V (green) and PI (red) positive signals in colorectal cancer cells but not in the normal colon epithelial cells. Additional deal death analyses on other cancerous and normal cells can be seen in Supplementary Figs 1 and 2, where similar findings of cancer selectivity were observed.

### Early Mitochondria Changes and Apoptotic Induction by Compounds A and I are Mediated by Caspase Dependent and Independent Mechanisms

**Figure 4:**
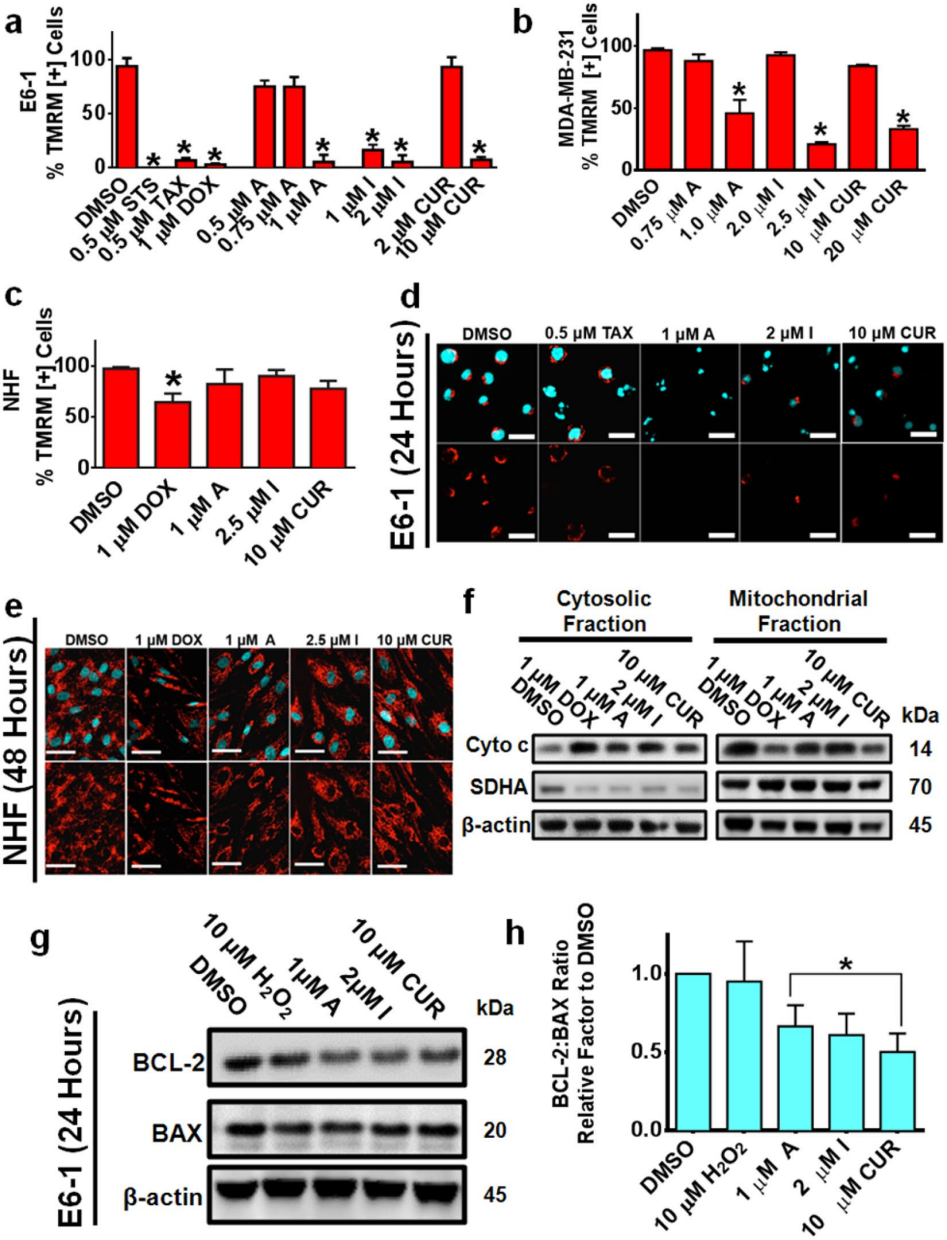
Selective Mitochondria Destabilization Following Treatment with Curcumin Analogs. (**a–c**) The cancerous cell lines E6-1 and MBA-MD-231 and normal cell line NHF were treated for 48 hours. To monitor mitochondria potential cells were incubated with TMRM for 45 minutes before analysis. Results were obtained using image-based cytometry with the Y-axis representative of percent of cells positive for TMRM expressed as a mean ± SD from three independent experiments. (**d**) Fluorescent microscopy images of acute T-cell leukemia (E6-1) cells stained with TMRM (red) and Hoechst (cyan) at 24 hours. Scale bar 25 microns. (**e**) Fluorescent microscopy images of NHF stained with TMRM (red) and Hoechst (cyan) at 48 hours. Scale bar 50 microns. Images are representative of three independent trials. (**f**) E6-1 cells were treated for 24 hours, following which the cytoplasmic and mitochondria fractions were separated and subjected to Western blot analysis. (**g**) E6-1 cells were treated for 24 hours, following which cells were lysed and subjected to Western blot analysis. Bands were visualized using a chemiluminescent reagent. (**h**) Densitometry results were obtained using Image J and are shown as a ratio of BCL-2 to BAX following normalization to β –actin. Results are representative of three independent trials. Statistical calculations were performed using One-Way ANOVA multiple comparison. *p < 0.05 vs Control (DMSO).

Apoptotic induction has been shown to be after mitochondria depolarization and induction of caspases. To monitor the mitochondrial stability and depolarization, a fluorescent TMRM assay was utilized as described in the materials and methods. Compounds A and I effectively decreased the percent of cancer cells positive for TMRM, indicating collapse of mitochondria potential at much lower concentrations than natural curcumin (Fig. 4a,b). Importantly, this effect was not observed in the normal human fibroblast (NHF) cells treated with equivalent doses of Compounds A and I (Fig. 4c). These results were further confirmed with fluorescent micrographs where limited TMRM staining in the E6-1 cell line at 24 hours was observed following treatment with Compound A and I, while there was punctate TMRM staining in the control and treated NHF cells at 48 hours (Fig. 4d,e). Similar decrease in TMRM positive cells for HCT-116 and MV-4-11 cell lines was observed with limited effects seen in the normal colorectal cell line, NCM460 (Supplementary Fig. 3). The destabilization of the mitochondria was further confirmed by monitoring the release of cytochrome c (Cyto C) into the cytosol following Compound A and I treatment using Western blot analysis of proteins in the mitochondria and cytosol: there was an increase in the levels of cytosolic Cyto C and a decrease in the mitochondrial fractions (Fig. 4f).

The ratio of anti-apoptotic BCL-2 to pro-apoptotic BAX protein plays a critical role in the survival and death of the cells^26, 27^. We monitored the level of BCL-2 to BAX proteins in E6-1 cells following treatment with Compound A and I. There was a decrease in the ratio of anti-apoptotic protein BCL-2 to the pro-apoptotic BAX protein (Fig. 4g,h) indicating a state that favors mitochondrial depolarization and subsequent execution of apoptosis.

**Figure 5:**
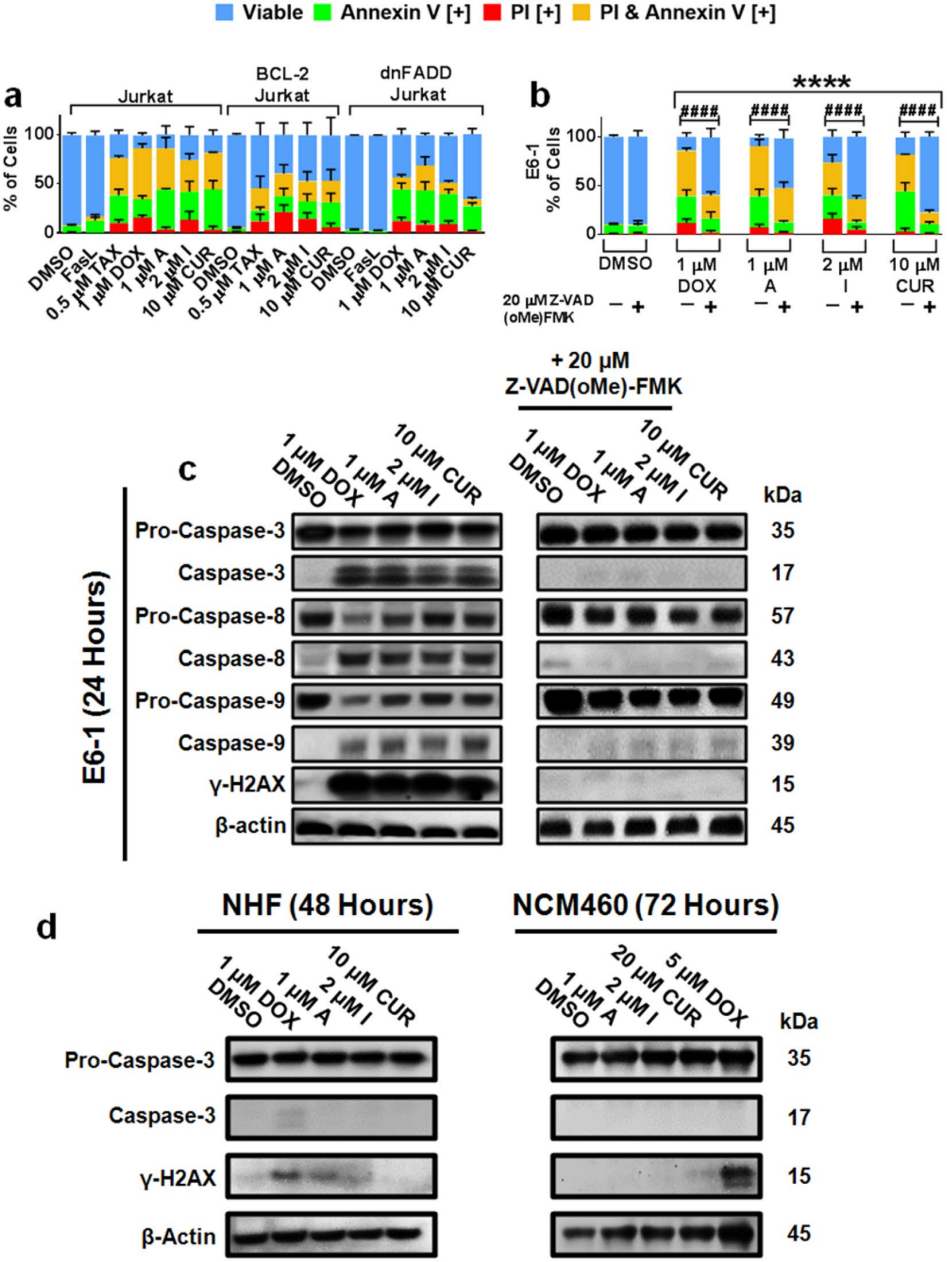
Curcumin Analogs Induce Apoptosis in Cancerous Cells by Several Pathways. (**a**) E6-1 (Jurkat), dominant negative FADD (dnFADD) Jurkat, and overexpressing BCL-2 Jurkat were treated for 48 hours then stained for Annexin V and PI (**b**) E6-1 cells were plated and treated with or without the broad spectrum caspase inhibitor ZVAD(oMe)-FMK for 48 hours. Cells were stained for Annexin V and PI. Results were obtained using image-based cytometry with the Y-axis representative of percent of cells positive for Annexin V (green), PI (red), Annexin V and PI (yellow), or negative for both Annexin V and PI (blue). Values are expressed as a mean ± SD from three independent experiments. (**c**) E6-1 cells were treated for 24 hours with or without the broad spectrum caspase inhibitor ZVAD(oMe)-FMK and the studied compounds, lysed and subjected to Western blot analysis. (**d**) NHF and NCM460 cells were treated for 48 hours and 72 hours respectively, lysed and subjected to Western blot analysis. Bands were visualized with a chemiluminescence reagent. Images are representative of three independent experiments. Statistical calculations were performed using Two-Way ANOVA multiple comparison. *p < 0.05 vs % viable of Control (DMSO); ^#^ *p* < 0.05 vs % viable cells for groups without Z-VAD(oMe)FMK.

Furthermore, to investigate the role of multiple death pathways, we utilized Jurkat cells either overexpressing BCL-2 (BCL-2 Jurkat) or lacking a functional Fas-associated protein with Death Domain (FADD; dnFADD Jurkat). Confirmation of a non-functional FADD protein can be seen by the lack of Annexin V and PI markers (Fig. 5a) and lack of caspase-8 cleavage (Supplementary Fig. 5) compared to the normal jurkat cells following treatment with the Fas ligand (FasL)^28^. Compounds A and I maintained their ability to induce apoptosis, albeit at a lower rate compared to the normal jurkat cell line (Fig. 5a). Interestingly, they maintained their ability to cause mitochondria destabilization in the BCL-2 Jurkat cell line (Supplementary Fig. 4a). Furthermore, when curcumin analogs and curcumin were added in combination with electron transport chain complexes I, II, or III inhibitors, there was no inhibition of apoptosis (Supplementary Fig. 4b). This indicates their activity to be independent of these complexes and the overall function of the electron transport chain.

To investigate the role of caspases in activation of apoptosis by Compound A and I, the broad-spectrum caspase inhibitor, Z-VAD(oMe)-FMK was utilized. In the presence of this inhibitor, there is a partial reduction in Annexin V and PI markers at 48 hours in E6-1 and MV-4-11 cell lines (Fig. 5b and Supplementary Fig. 6). Western blot analysis indicated that there was activation of caspase-8, -9, and -3 as well as increases in double-stranded DNA breaks (as indicated by γH2AX) following treatment with Compound A and I (Fig. 5c). However, Z-VAD(oMe)-FMK pre-treatment was able to effectively block the generation these markers. Moreover, the caspase-3 activation and presence of γH2AX were not evident in non-cancerous cells including NHF and NCM-460 following treatment with Compounds A and I for 48 or 72 hours (Fig. 5d), further supporting the selective nature of these analogs.

### Induction of Oxidative Stress by Compound A and I Selectively in Cancerous Cells is Critical for the Induction of Cell Death

**Figure 6:**
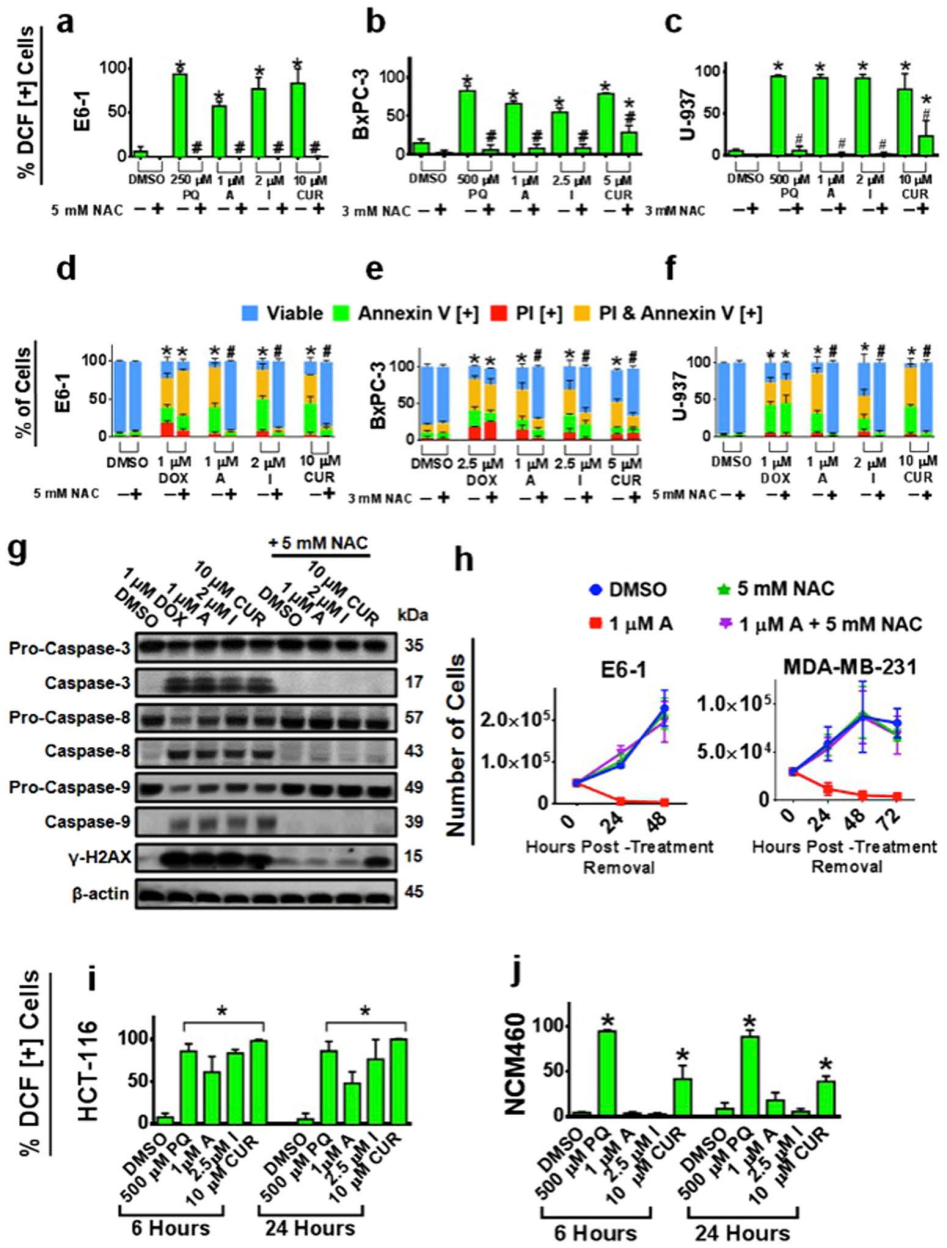
Curcumin Analogs are Dependent on the Production of Oxidative Stress to Induce Apoptosis. (**a–c**) Blood cancer (E6-1 and U-937) and pancreatic cancer (BxPC-3) cell lines were treated with H_2_DCFDA following treatments with or without the antioxidant, NAC and the studied compounds for 3 hours. Results were obtained using the image-based cytometry with the Y-axis representative of percent of cells positive for DCF. (**d–f**) Cancerous cell lines were treated with or without the antioxidant NAC for 48 hours. Following treatment, cells were stained for Annexin V and PI. Results were obtained using image-based cytometry with the Y-axis representative of percent of cells positive for Annexin V (green), PI (red), Annexin V and PI (yellow), or negative for both Annexin V and PI (blue). (**g**) E6-1 cells were treated for 24 hours with or without NAC and the studied compounds, lysed and subjected to Western blot analysis. Bands were visualized with a chemiluminescence reagent. (**h**) Cells were treated with the studied compounds for 24 or 48 hours respectively. Treatments were then removed and each group was replaced with media. Following 24, 48, or 72 hours, cells were counted using trypan blue. (**i**–**j**) Colorectal cancerous cells (HCT-116) and normal colon cells (NCM460) were treated with the studied compounds and monitored for the production of ROS at 6 and 24 hours using H_2_DCFDA. Results were obtained using the image-based cytometry with the Y-axis representative of percent of cells positive for DCF. All graphical values are expressed as a mean ± SD from three independent experiments. Statistical calculations were performed using Two-Way ANOVA multiple comparison for (**d-f**) and One-Way ANOVA multiple comparison for **(a–c**,**i–j**). *p < 0.05 vs % viable or DCF [+] cells of Control (DMSO); ^#^ *p* < 0.05 vs % viable or DCF [+] cells of groups treated without NAC.

Further investigating the mechanism of induction of apoptosis by these compounds, we observed that they induced production of ROS in different cancer cell lines as indicated by an increase in percent of cells positive for DCF relative to the control (Fig. 6a–c). We wanted to investigate if this increase in oxidative stress is essential in the downstream effects of these compounds for the induction of apoptosis by these analogs. When pre-treated with the antioxidant NAC, there was a near complete reduction in the markers for apoptosis and mitochondria destabilization across numerous cancerous cell lines following treatment with Compound A and I (Fig. 6d–f and Supplementary Fig. 7). Interestingly, Compounds A and I were able to induce ROS generation appreciably in the cancer colorectal cell line, HCT-116, but not in the normal NCM460, colon epithelial cells at 6 and 24 hours (Fig. 6i,j). Furthermore, these results were complimented by the reduction in the cleavage of caspase-3, -8, and -9 and presence of γH2AX (Fig. 6g) by NAC co-treatment.

As indicated above, the induction of apoptosis was near completely blocked as shown by the drastic decrease of apoptotic markers. However, further experiments were performed to determine if these cells rescued by NAC are fully functional in terms of cell division. Cells were treated with NAC, Compound A, or a combination of the two for 24 or 48 hours. Following the treatment, cells were washed and re-plated with an equal amount of cells in fresh media and counted at 24, 48, and 72 hours to obtain the growth kinetics (Fig. 6h). Compound A-treated groups were stagnant or decreased in total cell number for both MDA-MB-231 and E6-1 cell lines as time progressed, while the NAC and a combination of Compound A and NAC treated groups had a similar rate of growth to the DMSO (control) group.

Previous work has shown a water-soluble formulation of Coenzyme Q_10_ (Ubs-CoQ_10_) to inhibit ROS production and protect against mitochondria-mediated apoptosis^29, 30^. Ubs-CoQ_10_ was used and shown to reduce the percent of cells that underwent apoptosis by Compound A and I as indicated by the reduction in the Annexin V and PI apoptotic markers (Supplementary Fig. 8) further supporting the role of oxidative stress caused by these compounds in inducing cancerous cell death.

### Gene Expression Analysis of Oxidative Stress-Related Genes in Cancer and Normal Colon Cell Lines Following Treatment with Compound A

**Figure 7:**
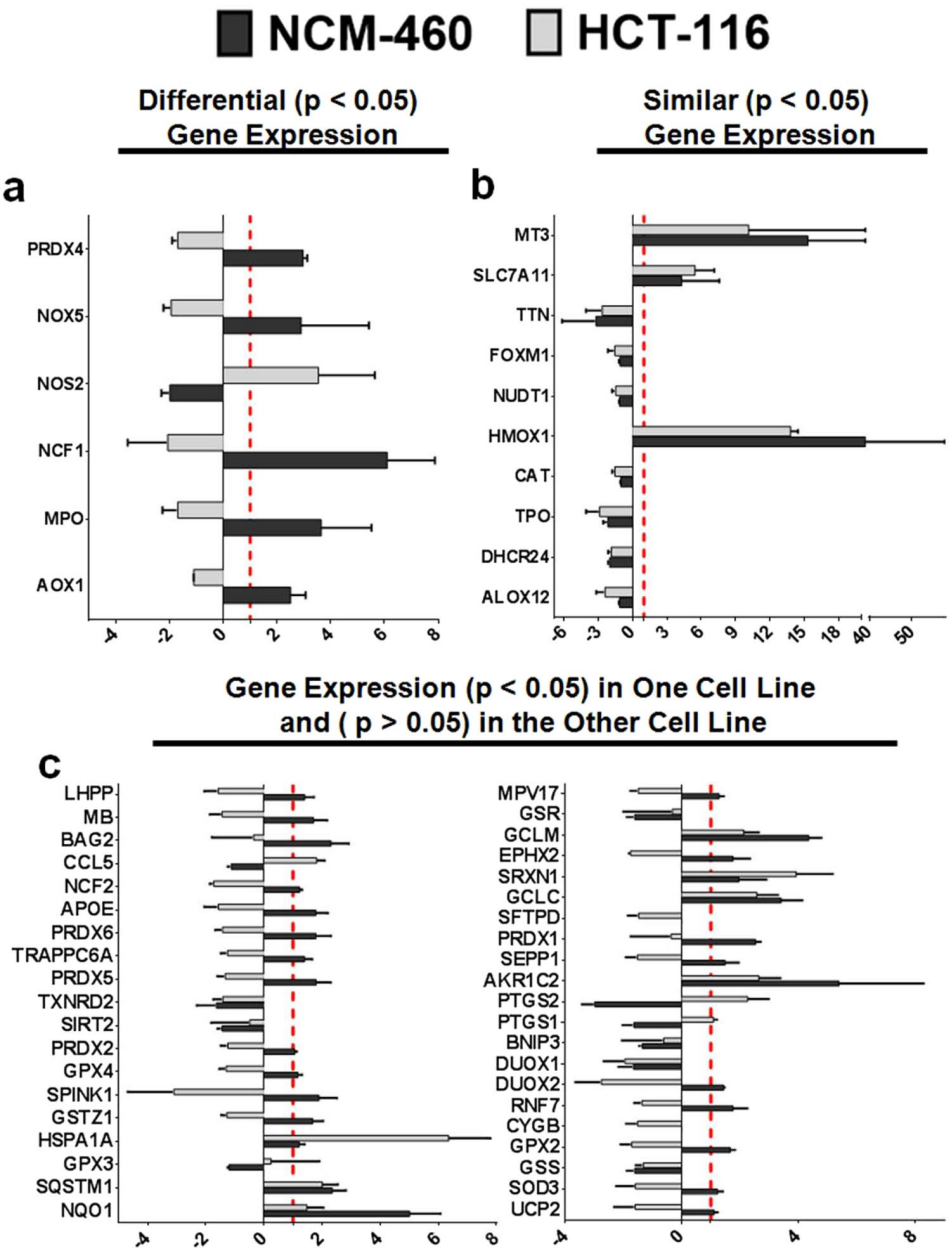
Comparative Change in Oxidative Stress-Related Gene Expression Between Cancerous and Non-Cancerous Cell Lines. The cancerous cell line (HCT-116) and non-cancerous cell line (NCM-460) were treated with the vehicle control (DMSO) or 1 µM Compound A for 6 hours. RNA isolation and subsequent RT^2^ PCR analysis was carried out. Results are shown as a fold-change relative to control following treatment with Compound A of three independent trials. Y-axis: Gene. X-axis: Fold-Change to control. The red dashed line is set at a relative fold-change of one, representing the control.

To investigate if the treatment with Compound A leads to differential gene expression profile in cancer cells compared to normal cells, RT^2^ PCR was carried out. Since an increase in ROS production was the first event observed at 6-hours following the treatment with no significant cell death, analysis was performed at this time point (Fig. 6i and Supplementary Fig. 9). In summary, of the 84 genes analyzed, six genes were clearly differentially expressed between cancer and non-cancerous cell types (Fig. 7a). These genes are involved in maintaining redox homeostasis, production of reactive oxygen species, and inflammatory signaling. Five of these (AOX1, MPO, PRDX4, NCF1, and NOX5) were found to be decreased in the cancerous cells following Compound A treatment while these genes were up-regulated in the normal cell line. These observations indicate that cancer cells may respond in exact opposite way in terms of gene expression to Compound A treatment. Furthermore, following treatment with Compound A, there were over 40 genes related to radical quenching, oxidative stress response, and autophagy or inflammatory pathways, which had a statistically significant change in mRNA expression in one cell line and a non-significant change in the other (Fig. 7c). These are important observations, which indicated that cancer and normal cells trigger differential gene expression response following Compound A treatment.

Ten genes were found to have similar pattern of statistically significant changes in mRNA expression in both cell types (Fig. 7b) including a decrease in genes related to cell proliferation, migration (ALOX12 and FOXM1), ROS quenching proteins (CAT and TPO) as well as an increase in genes involved in preventing oxidative stress or cell death (MT3 and HMOX1).

Lastly, the other twenty-eight genes analyzed were found to be statistically non-significant in their change in mRNA expression and can be found in Supplementary Fig. 10. A summary of all genes, their abbreviated function, relative-fold change, and statistical significance can be found in Tables 2–8.

**Table 2:** Summary of the Relative-Fold Change in Gene Expression Utilizing RT^2^ PCR.

		Gene Function	Fold Change (±STDEV)	
Gene			HCT-116	NCM-460
Contrast Gene Expression Change (*p* < 0.05)	AOX1 (Aldehyde Oxidase 1)	Is an oxidase invovled in ROS homeostasis and/or metabolism of certain xenobiotics and drugs.	−1.10 (0.04)*	2.51 (0.55)*
	MPO (Myeloperoxidase)	Produces hypohalous acids, such as hypocholorous acid (HClO)	−1.69 (0.57)**	3.64 (1.87)***
	NOS2 (Nitric oxide synthase 2)	Production of nitric oxide (NO), which is invovled in the inflammation process, and augments pro-inflammatory signaling.	3.54 (2.10)**	−1.98 (0.32)****
	PRDX4 (Peroxiredoxin 4)	Invovled in redox signaling, and reducing peroxides through the thioredoxin system	−1.69 (0.21)**	2.97 (0.16)**
	NCF1 (Neutrophil cytosolic factor 1)	Membrane bound protein involved in the activation of NADPH oxidase leading to superoxide production	−2.07 (1.49)**	6.09 (1.78)****
	NOX5 (NADPH oxidase, EF-hand calcium binding domain 5)	Calcium-dependent NADPH oxidase, invovled in production of superoxidee radicals, affecting cell growth and metabolism.	1.94 (0.29)**	2.90 (2.52)*

Statistical analysis was performed using One-Way ANOVA to relative-fold change of the control (1). *p < 0.05 vs control; **p < 0.01 vs control; ***p < 0.001 vs control; ****p < 0.0001 vs control. n.s. = not significant, *p* > 0.05 vs control. N/A = not applicable due to no amplification detected. Gene function was obtained searching the UniProt or NCBI Gene Databases.

**Table 3:** Summary of the Relative-Fold Change in Gene Expression Utilizing RT^2^ PCR.

		Gene Function	Fold Change (±STDEV)	
Gene			HCT-116	NCM-460
Similar Gene Expression Change (*p* < 0.05)	ALOX12 (Arachidonate 12-lipoxygenase)	Iron-containing enzyme, may play a role in apoptosis, cell proliferation and migration.	−2.40 (0.78)***	−1.13 (0.07)**
	DHCR24 (Dehydrocholesterol reductase)	Invovled in reduction of sterol intermediates. Potentially protects cells from apoptosis induced by oxidative stress by decreasing caspase-3 activity.	−1.85 (0.27)**	−1.66 (0.51)****
	TPO (Thyroid peroxidase)	Invovled in thyroid hormone T_3_ and T_4_ production. Oxidizes iodide using hydrogen peroxide.	−2.88 (1.17)***	−2.14 (0.43)****
	CAT (Catalase)	Quenches hydrogen peroxide into molecular oxygen and water.	−1.54 (0.23)**	−1.03 (0.02)**
	HMOX1 (Heme oxygenase 1)	Invovled in heme catabolism and anti-apoptotic properties	13.82 (0.65)****	35.75 (21.96)****
	NUDT1 (Nucleoside diphosphate linked moiety X (nudix)-type motif 1)	Acts as an anti-mutagenic agent by suppressing oxidized nucleotides reducing death induced through oxidative stress pathways.	−1.48 (0.31)**	−1.13 (0.04)**
	FOXM1 (Forhead box M1)	Transcription factor regulating cell cycle genes essential for cell division. May play a role in proliferation and DNA damage response system.	−1.55 (0.60)**	−1.32 (0.30)***
	TTN (Titin)	Plays a role in chromosomal condensation and segregation during mitosis in non-muscle cells.	−2.65 (1.41)***	−3.19 (2.96)****
	SLC7A11 (Solute carrier family 7, member 11)	Sodium-independent amino acid transporter, with high affinity for the anionic forms of amino acids cysteine and glutamate.	5.47 (1.67)****	4.30 (3.29)****
	MT3 (Metallothionein 3)	Invovled in heavy metal binding, preventing from oxidative stress and suspected to play a role in resistance to certain chemotherapeutics.	10.20 (10.30)****	15.32 (7.28)****

Statistical analysis was performed using One-Way ANOVA to relative-fold change of the control (1). *p < 0.05 vs control; **p < 0.01 vs control; ***p < 0.001 vs control; ****p < 0.0001 vs control. n.s. = not significant, *p* > 0.05 vs control. N/A = not applicable due to no amplification detected. Gene function was obtained searching the UniProt or NCBI Gene Databases.

**Table 4:** Summary of the Relative-Fold Change in Gene Expression Utilizing RT^2^ PCR.

		Gene Function	Fold Change (±STDEV)	
Gene			HCT-116	NCM-460
Gene Expression Change (*p* < 0.05) Only in one Cell Line	NQO1 (NAD(P)H dehydrogenase, quinone 1	Reduces quinone to hydroquinone via NAD(P)H.	1.48 (0.56) n.s.	5.02 (1.06)****
	SQSTM1 (Sequestosome 1)	Autophagy receptor binding to cargo and LC3-like proteins.	2.01 (0.53) n.s.	2.35 (0.47)*
	GPX3 (Glutathione peroxidase 3)	Protects cells from oxidative damage by reduction of peroxides using glutathione.	0.25 (1.65) n.s.	−1.20 (0.04)**
	HSPA1A (Heat shock 70 kDa protein 1 A)	Acting as a chaperon protein in the cytosol and within organelles.	6.36 (1.43)****	2.15 (0.98) n.s.
	GSTZ1 (Glutathione S-transferase zeta 1)	Serves as a glutathione-conjugating and -peroxidase enzyme.	−1.27 (0.20)*	1.22 (0.18) n.s.
	SPINK1 (Serine Peptidase inhibitor, kazal type 1)	A serine protease inhibitor, displaying anti-trypsin activity which defends against untimely trypsin-catalyzed activation of zymogens.	−3.10 (1.59)****	1.90 (0.62) n.s.
	GPX4 (Glutathione peroxidase 4)	Involved in the production glutathione disulfide, playing a protective role against radiation and oxidative damage.	−1.30 (0.23)*	1.17 (0.14) n.s.
	PRDX2 (Peroxiredoxin 2)	Involved in redox signaling, and reducing peroxides through the thioredoxin system.	−1.24 (0.24)*	1.06 (0.07)
	SIRT2 (Sirtuin 2)	Exact function remains to be elucidated but may regulate proteins with ADP-ribosyltransferase activity.	−0.48 (0.33) n.s.	−1.44 (0.15)**
	TXNRD2 (Thioredoxin reducatase 2)	Maintains thioredoxin in reduced state, protecting mitochondria from oxidative stress. May play a role in redox-regulated cell signaling.	−1.41 (0.33) n.s.	−1.63 (0.68)****
	PRDX5 (Peroxiredoxin 5)	Invovled in redox signaling and reducing peroxides through the thioredoxin system.	−1.33 (0.27)**	1.80 (0.50) n.s.
	TRAPPC6A (Trafficking protein particle complex 6A)	Plays a role in vesicular transport during melanosome biosynthesis.	−1.25 (0.23)*	1.40 (0.27) n.s.
	PRDX6 (Peroxiredoxin 6)	Involved in redox signaling, and reducing peroxides through the thioredoxin system.	−1.42 (0.24)**	1.79 (0.51) n.s.

Statistical analysis was performed using One-Way ANOVA to relative-fold change of the control (1). *p < 0.05 vs control; **p < 0.01 vs control; ***p < 0.001 vs control; ****p < 0.0001 vs control. n.s. = not significant, *p* > 0.05 vs control. N/A = not applicable due to no amplification detected. Gene function was obtained searching the UniProt or NCBI Gene Databases.

**Table 5:** Summary of the Relative-Fold Change in Gene Expression Utilizing RT^2^ PCR.

		Gene Function	Fold Change (±STDEV)	
Gene			HCT-116	NCM-460
Gene Expression Change (*p* < 0.05) Only in one Cell Line	DUOX2 (Dual oxidase 2)	Generates hydrogen peroxide.	−2.76 (0.89)****	1.16 (0.14) n.s.
	DUOX1 (Dual oxidase 1)	Generates hydrogen peroxide.	−1.95 (0.73)***	1.44 (0.03) n.s.
	BNIP3 (BCL2/adenovirus E1B 19 kDa interacting protein 3)	Apoptosis-indcuing protein that can overcome BCL-2 suppression.	−0.62 (1.43) n.s.	−1.35 (0.11)***
	PTGS1/COX-1 (Prostaglandin-endoperoxide synthase 1)	Produces prostanoids and plays a role in cytoprotection.	1.09 (0.12)	−1.64 (0.38)***
	PTGS2/COX-2 (Prostaglandin-endoperoxide synthase 2)	Invovled in inflammatory responses, found to be constitutively expressed in some endothelial cancers.	2.26 (0.73) n.s.	−2.98 (0.44)****
	AKR1C2 (Aldo-keto reducase family 1, member C2)	Converts steroid hormones by reactions with NADP^+^.	2.75 (0.74) n.s.	5.40 (2.90)****
	SEPP1 (Selenoprotein P, plasma 1)	May regulate the extracellular antioxidant defense characteristics of selenium. May aid in selenium transport.	−1.52 (0.39)**	1.30 (0.17) n.s.
	PRDX1 (Peroxiredoxin 1)	Plays a role in redox-regulation of the cell by regerating glutathione levels.	−0.36 (1.39) n.s.	2.54 (0.17)*
	SFTPD (Surfactant protein D)	Involved in pulmonary defenses against inhaled microorganisms and toxins. Can alter the action of leukocytes in immune response, through negative regulation of interleukin-2 (IL-2) cytokine signaling.	−1.48 (0.36)**	N/A
	GCLC (Glutamate-cyteine ligase, catalytic subunit)	Catalyzes glutamate and cysteine using ATP.	2.58 (0.73) n.s.	3.41 (0.72)***
	SRXN1 (Sulfiredoxin 1)	Involved in oxidative stress resistance through reduction of cysteine-sulfinic acid on PRDX1-4 under exposure to oxidants. Does not act on PRDX5/6.	3.93 (1.25)***	1.94 (0.94) n.s.
	EPHX2 (Epoxide hydrolase 2)	Plays a role in xenobiotic metabolism by catalyzing epoxides with water to produce a diol	−1.75 (0.05)**	1.06 (0.04) n.s.
	GCLM (Glutamate-cysteine ligase, modifier subunit)	Regulates biosynthetic pathway of glutathione by modulating GCLC activity.	2.13 (0.51) n.s.	4.36 (0.43)****
	GSR (Glutathione Reductase)	Maintains reduced glutathione levels in the cytosol.	−0.32 (1.69) n.s.	−1.61 (0.27)***

Statistical analysis was performed using One-Way ANOVA to relative-fold change of the control (1). *p < 0.05 vs control; **p < 0.01 vs control; ***p < 0.001 vs control; ****p < 0.0001 vs control. n.s. = not significant, *p* > 0.05 vs control. N/A = not applicable due to no amplification detected. Gene function was obtained searching the UniProt or NCBI Gene Databases.

**Table 6:** Summary of the Relative-Fold Change in Gene Expression Utilizing RT^2^ PCR.

		Gene Function	Fold Change (±STDEV)	
Gene			HCT-116	NCM-460
Gene Expression Change (*p* < 0.05) Only in one Cell Line	APOE (Apolipoprotein E)	Involved in lipoprotein particle trafficking.	−1.57 (0.49)**	2.51 (0.55) n.s.
	NCF2 (Neutrophil cytosolic factor 2)	Invovled in the activation of NADPH oxidase leading to superoxide production.	−1.73 (0.13)**	1.23 (0.08) n.s.
	CCL5 (Chemokine, c-c motif ligand 5)	Plays a role in chemotaxis and several cell signaling pathways.	1.81 (0.27) n.s.	−1.12 (0.12)**
	BAG2 (BCL-2 associated athanogene 2)	Chaperone protein involved in several pro-apoptotic pathways	−0.35 (1.43) n.s.	2.31 (0.61)*
	MB (Myoglobin)	Involved in oxygen binding and storage.	−1.44 (0.41)*	1.71 (0.47) n.s.
	LHPP (Phospholysine phosphohistidine inorganic pyrophosphate phosphatase)	Possesses phosphatase activity. Hydrolyzes imidodiphosphate, 3-phosphohistidine and 6-phospholysine.	−1.57 (0.47)**	1.40 (0.32) n.s.
	MPV17 (MpV17 mitochondrial inner membrane protein)	Potentially invovled with the metabolism of ROS and homeostasis of oxidative phosphorylation and mitochondria DNA	−1.49 (0.27)*	1.28 (0.16) n.s.
	UCP2 (Uncoupling protein 2)	Uncouples oxidative phosphorylation from ATP generation by dissipating the proton gradient across the inner mitochondrial membrane, resulting in heat production.	−1.59 (0.72)**	1.11 (1.12) n.s.
	SOD3 (Superoxide dismutase 3)	Involved in quenching extracellular superoxide radicals, converting these radicals to hydrogen peroxide and oxygen.	−1.59 (0.65)**	1.23 (0.19) n.s.
	GSS (Glutathione synthetase)	Involved in glutathione biosynthesis	−1.32 (0.26)*	1.29 (0.08) n.s.
	GPX2 (Glutathione peroxidase 2)	Involved in quenching of ROS utilizing glutathione.	−1.71 (0.38)*	1.67 (0.16) n.s.
	CYGB (Cytoglobin)	May be involved in storage or transfer of oxygen as well as a protective function during oxidative stress.	−1.51 (0.40)**	N/A
	RNF7 (Ring finger protein 7)	Component of the SKP1-CUL1-F-box protein complex involved in ubiquitination, and subsequent proteasomal degradation of proteins involved in cell cycle regulation.	−1.36 (0.27)**	1.76 (0.49) n.s.

Statistical analysis was performed using One-Way ANOVA to relative-fold change of the control (1). *p < 0.05 vs control; **p < 0.01 vs control; ***p < 0.001 vs control; ****p < 0.0001 vs control. n.s. = not significant, *p* > 0.05 vs control. N/A = not applicable due to no amplification detected. Gene function was obtained searching the UniProt or NCBI Gene Databases.

**Table 7:** Summary of the Relative-Fold Change in Gene Expression Utilizing RT^2^ PCR.

		Gene Function	Fold Change (±STDEV)	
Gene			HCT-116	NCM-460
Non-significant Gene Expression Change (*p* > 0.05) in Both Cell Lines	TXN (Thioredoxin)	Invovled in oxidation-reduction reactions of sulfide groups.	−0.38 (0.23) n.s.	2.26 (0.75) n.s.
	ALB (Albumin)	Binds several metals and molecules.	1.74 (0.51) n.s.	2.02 (0.90) n.s.
	FHL2 (Four and a half LIM domains 2)	Inhibits transcriptional activity of cell growth transcription factor FOXO1.	0.34 (1.24) n.s.	1.76 (0.18) n.s.
	SOD1 (Superoxide dismutase 1)	Quenches hydrogen peroxide.	0.46 (1.34) n.s.	2.19 (0.74) n.s.
	PRNP (Prion protein)	Unclear function but suspected to play a role in iron homeostasis.	0.54 (1.42) n.s.	1.54 (0.04) n.s.
	VIMP (VCP-interactin membrane protein)	Involved in degradation process of misfolded proteins in the endoplasmic recticulum.	−0.51 (1.36) n.s.	N/A
	FTH1 (Ferritin, heavy polypeptide 1)	Transporter of iron.	−0.23 (1.44) n.s.	2.10 (0.64) n.s.
	MBL2 (Mannose-binding lectin (protein C) 2)	Binds to mannose and related molecule, may play a role in binding to late apoptotic cells facilitating uptake by macrophages.	N/A	N/A
	MSRA (Methionine sulfoxide reductase A)	Catalyzes the oxidation-reduction of methionine sulfoxide to methionine in proteins.	−0.60 (1.93) n.s.	1.37 (0.09) n.s.
	SOD2 (Superoxidde dismutase 2)	Invovled in quenching hydrogen peroxide	−0.78 (1.60) n.s.	1.55 (0.31) n.s.
	CCS (Copper chaperone for superoxide dismutase)	Delivers copper to SOD1.	−0.28 (1.46) n.s.	1.13 (0.11) n.s.
	GSTP1 (Glutathione S-transferase pi 1)	Conjugates reduced glutathione to various biomolecules	−0.33 (1.43) n.s.	1.68 (0.36) n.s.
	PTGR1 (Prostaglandin reductase 1)	Reduces 15 carbon long alkanal molecules to alk-2-enals.	−0.62 (1.42) n.s.	1.71 (0.23) n.s.
	HSP90AA (Heat shock protein 90 kDa alpha (cytosolic), class A member 1)	Chaperone protein, important in maturation and structural maintenance of a variety of proteins.	1.37 (0.48) n.s.	2.25 (0.37) n.s.
	PRDX3 (Peroxiredoxin 3)	Involved in redox homeostasis. Located in the mitochondria.	−0.70 (1.62) n.s.	1.99 (0.85) n.s.

Statistical analysis was performed using One-Way ANOVA to relative-fold change of the control (1). *p < 0.05 vs control; **p < 0.01 vs control; ***p < 0.001 vs control; ****p < 0.0001 vs control. n.s. = not significant, *p* > 0.05 vs control. N/A = not applicable due to no amplification detected. Gene function was obtained searching the UniProt or NCBI Gene Databases.

**Table 8:** Summary of the Relative-Fold Change in Gene Expression Utilizing RT^2^ PCR.

		Gene Function	Fold Change (±STDEV)	
Gene			HCT-116	NCM-460
Non-significant Gene Expression Change (*p* > 0.05) in Both Cell Lines	GLA (Galactosidase, alpha)	Hydrolyzes several sugars	1.98 (0.66) n.s.	2.17 (0.18) n.s.
	GPX1 (Glutathione peroxidase 1)	Quenches hydrogen peroxide using glutathione.	−0.67 (1.45) n.s.	1.64 (0.01) n.s.
	KRT1 (Keratin 1)	Primarily involved in hair differentiation	N/A	N/A
	ATOX1 (Antioxidant 1 copper chaperone)	Delivers cytosolic copper to ATPase proteins. Important in cellular antioxidant defenses	−0.64 (1.48) n.s.	1.73 (0.47) n.s.
	CYBB (Cytochrome b-245, beta polypeptide)	Involved in transfer of NADPH into the cytosol.	N/A	N/A
	PDLIM1 (PDZ and Lim domain 1)	Acts as an adapter protein for protein anchoring to cell membrane.	0.24 (1.54) n.s.	1.40 (0.15) n.s.
	NCOA7 (Nuclear receptor coactivator 7)	Enhances activity of nuclear receptors	0.52 (1.45) n.s.	1.40 (0.15) n.s.
	DUSP1 (Dual specificy phosphatase 1)	Dephosphorylates MAP kinase	1.57 (0.46) n.s.	1.76 (0.58) n.s.
	LPO (Lactoperoxiase)	Reacts with hydrogen peroxide to produce hypothiocyanous acid.	N/A	N/A
	GPX5 (Glutathione peroxidase 5)	Epididymis secretory glutathione peroxidase, protecting cells from oxidative stress using glutathione.	N/A	N/A

Statistical analysis was performed using One-Way ANOVA to relative-fold change of the control (1). *p < 0.05 vs control; **p < 0.01 vs control; ***p < 0.001 vs control; ****p < 0.0001 vs control. n.s. = not significant, *p* > 0.05 vs control. N/A= not applicable due to no amplification detected. Gene function was obtained searching the UniProt or NCBI Gene Databases.

### Apoptotic Induction of Compound A is Enhanced by Combinatorial Treatment with Piperlongumine, Another ROS Inducing Compound

**Figure 8:**
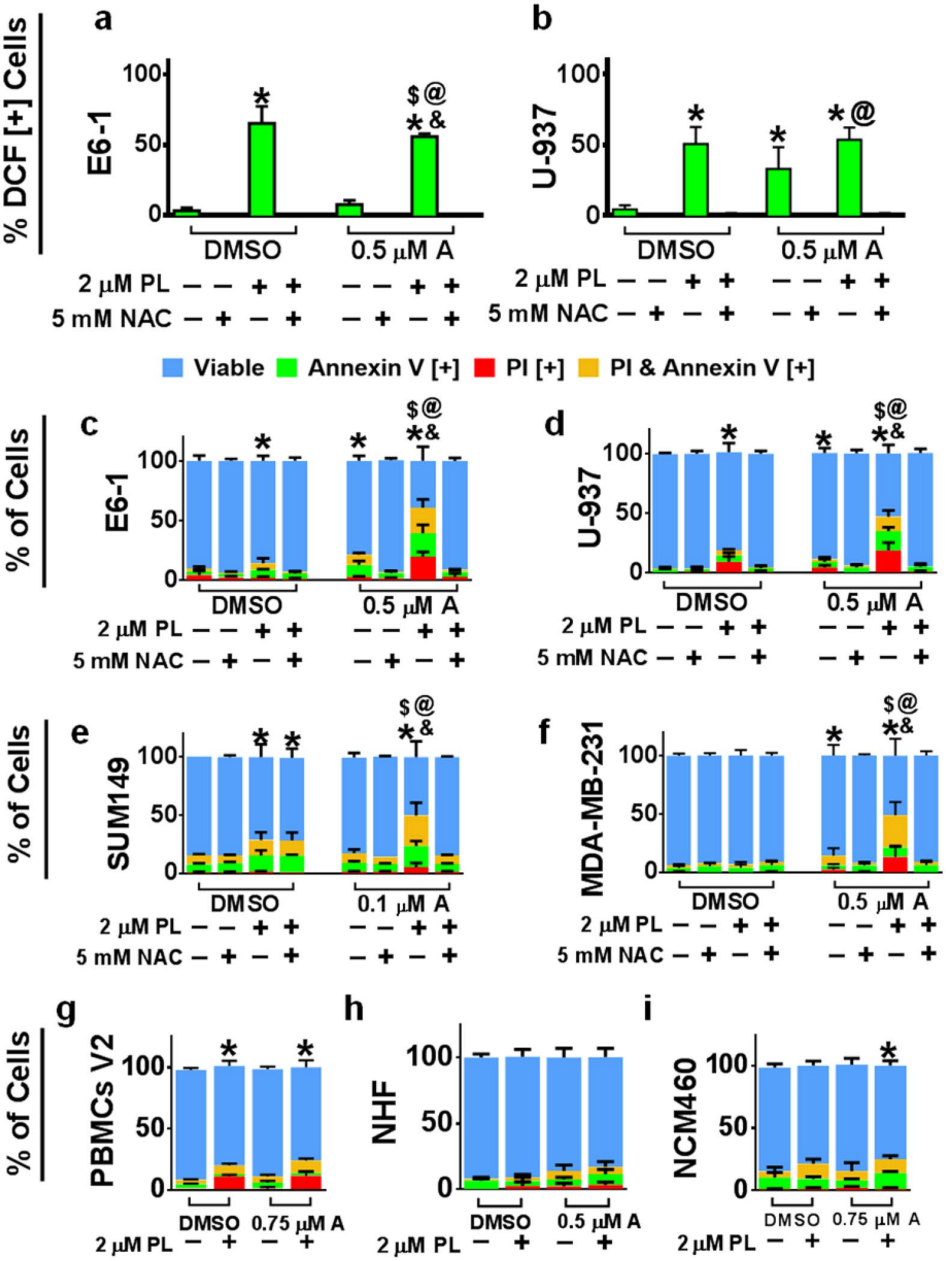
Synergistic Effect of Compound A and PL to Induce Apoptosis Selectively in a Variety of Cancer Cell Lines. (**a,b**) E6-1 and U-937 cell lines were treated with H_2_DCFDA then with Compound A either alone, in combination with NAC or PL alone, or both NAC and PL for 3 hours to monitor the production of ROS. Results were obtained using the image-based cytometry. The Y-axis is representative of percent of cells positive for DCF. (**c–f**) Triple-negative-breast cancer (SUM-149, MDA-MB-231) and blood cancer (E6-1, U-937) cell lines were treated with DMSO, Compound A either alone, in combination with NAC or PL alone, or both NAC and PL for 48 hours. Following treatment, cells were stained for Annexin V and PI. Results were obtained using image-based cytometry. (**g–i**) Peripheral mononuclear blood cells (PBMCs) acquired from a healthy volunteer, normal human fibroblast (NHF) and colon (NCM460) cells were treated with the alone or combinatorial doses for 48 hours. Following treatment, cells were stained for Annexin V and PI. Results were obtained using image-based cytometry. The Y-axis is representative of percent of cells positive for Annexin V (green), PI (red), Annexin V and PI (yellow), or negative for both Annexin V and PI (blue). All graphical values are expressed as a mean ± SD from three independent experiments. Statistical calculations were performed using Two-Way ANOVA multiple comparison (**a–d**,**g–i**) or One-Way ANOVA multiple comparison (**e,f**). *p < 0.05 vs % viable or DCF [+] cells of control (DMSO); ^@^ *p* < 0.05 vs % viable or DCF [+] cells of group treated with PL, NAC, and Compound A; ^&^ *p* < 0.05 vs % viable or DCF [+] cells of group treated with Compound A; ^$^ *p* < 0.05 vs % viable or DCF [+] cells of group treated with PL.

Recently, the natural compound piperlongumine (PL) has been found to induce ROS production and apoptosis selectively against cancer cells *in vitro* and *in vivo* taking advantage of their altered redox state^31^. We wanted to investigate if adding PL in combination with Compound A will have enhanced/synergistic effects in terms of inducing cell death in cancer cells. Indeed, PL was able to induce ROS generation in cancer cell lines alone (Fig. 8a,b), but in combination with Compound A there was a synergism in apoptotic induction in several blood (Fig. 8c,d) and triple-negative breast (Fig. 8e,f) cancer cell lines. More importantly, this combinatorial treatment was well tolerated in non-cancerous cells including PMBCs, NCM460, and NHF cells (Fig. 8g–i) exemplifying the selective anti-cancer activity of this combination treatment. These results were further confirmed by the absence of caspase-3 activation and presence of γH2AX in the NHFs at 48 hours (Supplementary Fig. 12) following the combinatorial treatment. Similar synergistic apoptotic and ROS generation effects were found in the BxPC-3 pancreatic cancer cell line (Supplementary Fig. 11).

### Intraperitoneal Administration of Compound A Effectively Suppresses Xenograft Growth of Human Leukemia and Triple-Negative Breast Cancer in Mice

**Figure 9:**
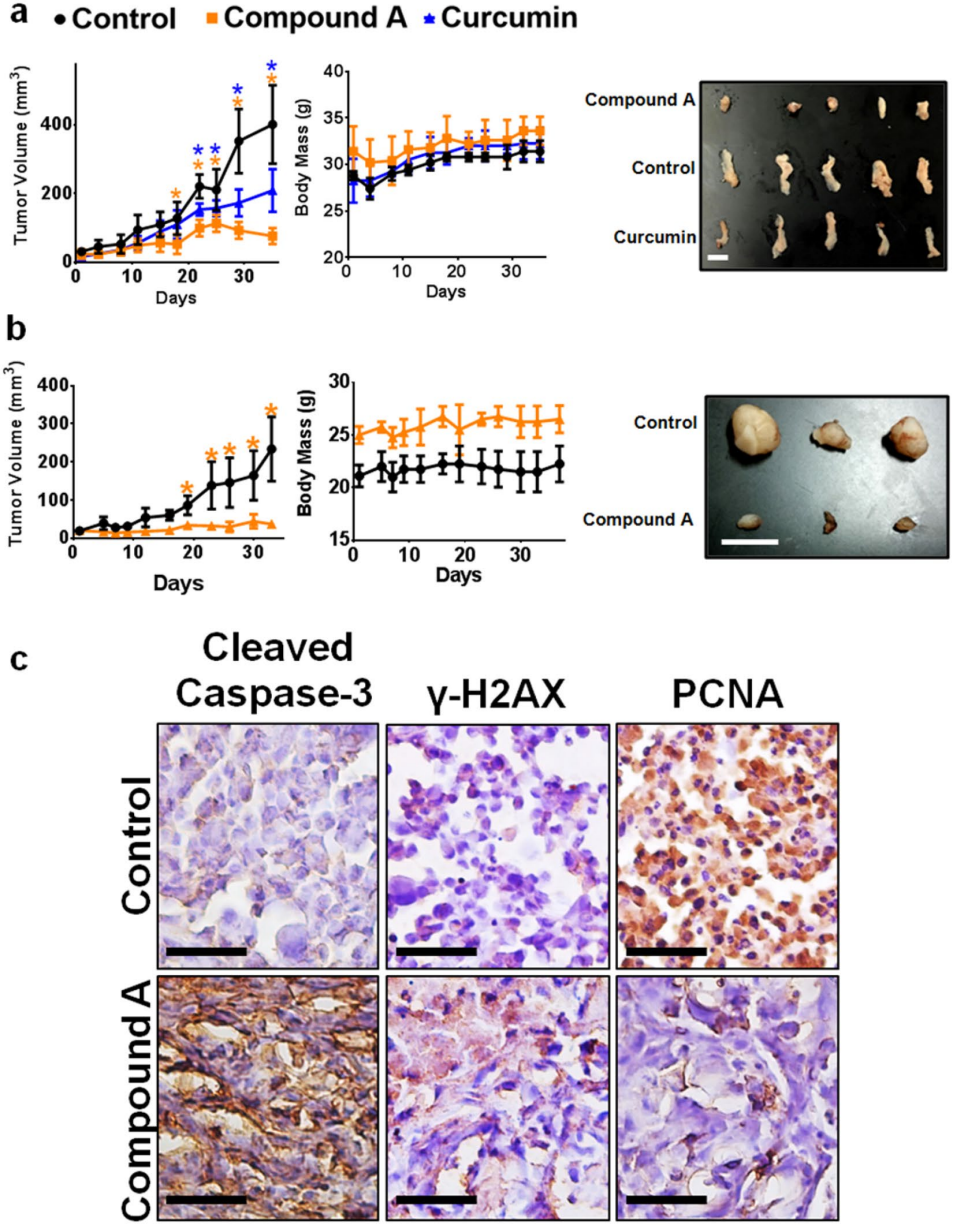
Intraperitoneally Administered Compound A Effectively Suppresses Triple-Negative Breast and Blood Cancer Xenograft Tumor Growth. Immunocompromised mice were subcutaneously injected with cancerous cell lines and tumors were allowed to establish. Treatments occurred every other day and the studied compound or the equivalent vehicle control administered intraperitoneally for five weeks. Tumor volume and mass were measured two times per week. (**a**) MV-4-11 xenograft study was administered with approximately 5.0 mg/kg of Compound A and 5.0 mg/kg of curcumin. Results are representative for the Control (n = 5), Compound A (n = 5), and curcumin (n = 4) treated group, each with two tumors per mouse. (**b**) MDA-MB-231 xenograft study was administered with approximately 7.5 mg/kg of Compound A. Results are representative for the Control (n = 3) and Compound A (n = 4), each with one tumor per mouse. Scale bar is 1 centimeter. (**c**) Immunohistochemistry analysis of sectioned tumor tissues from the MDA-MB-231 study. Each section was subjected to the specified antibody followed by a biotinylated secondary antibody. Detection was done using a DAB Peroxidase HRP Substrate Kit (brown) followed by Hematoxylin counterstaining (purple). Images were obtained using inverted bright field microscopy. Sectioning results are representative of three individual tumors. Scale bar is 50 microns. Statistical analysis using One-Way ANOVA. *p < 0.05 vs tumor volume of the control.

Having seen effective and selective induction of cell death by Compound A in a number of cancer cell lines, we wanted to evaluate if this compound and its ability to inhibit the growth of aggressive, triple-negative breast cancer and leukemia xenografts in mice. Human leukemia (MV-4–11) and triple-negative breast cancer (MDA-MB-231) cells were transplanted subcutaneously in immunocompromised mice as described in materials & methods. Following tumor establishment, Compound A was administered intraperitoneally over the course of 5 weeks. Compound A was able to decrease the growth of the xenograft as determined by tumor size relative to the vehicle control (Fig. 9a,b). Furthermore, in the MV-4-11 xenograft model, Compound A was slightly more effective at suppressing tumor growth compared to natural curcumin given at similar doses. Over the five-week studies, there was no apparent change in body weight of mice in each group compared to the control, indicating tolerance of the given compounds by the animals. Furthermore, immunohistochemical staining indicated apoptotic induction by Compound A in tumor tissues and not in vital organs and noncancerous mouse tissue (Fig. 9c). There was an increase in cleaved caspase-3 and γH2AX as well as a reduction in positive staining for PCNA, a marker for cellular proliferation, in the Compound A treated group compared to the control group in the tumor sections. Additionally, there was a reduction in VEGF in the tumor sections and no apparent changes in PCNA or γH2AX staining in heart and kidney sections respectively for both groups (Supplementary Fig. 14). Thus, these findings illustrate that Compound A is effectively in reducing tumor growth *in vivo* and is well tolerated by the animals used in this study.

## Discussion

Curcumin has been enticing for its use as a chemopreventative and chemotherapeutic compound. Previous literature has shown curcumin to be well tolerated in humans and to induce apoptosis selectively in cancerous cells with limited effects in normal cells *in vitro* ^32–34^. However, its limited bioavailability and stability has limited its further use in a clinical application^18, 19, 35^. Herein, we present two novel MACs that are more chemically stable and effective at inducing cell death in a variety of human cancer cells including notoriously resistant, triple-negative breast and p53-negative colorectal cancer cells at doses several-fold less than natural curcumin. Furthermore, we demonstrated that their selective targeting to cancer cells is attributed to their ability to induce oxidative stress selectively in cancer cells. One of the MACs, Compound A was effective in halting the growth of human leukemia and triple-negative breast cancer xenografts in mouse models when administered intraperitoneally, indicating they are absorbed and are stable within a physiological system.

In this body of work, several MACs have been synthesized and we screened 10 analogs, Compounds A-J (Fig. 1) for their cytotoxicity towards cancerous cells (Fig. 2 and Table 1). A detailed analysis of mode of cell death induction in cancer cells by two novel MACs, Compound A and I was carried out utilizing biochemical and morphological markers of apoptosis. Compounds A and I were shown to be potent inducers of apoptosis in different cancerous cells selectively (Fig. 3a–e and Supplementary Fig. 1e) with minimal effect in several normal cell lines tested (Figs 3f–j and 4c,e, Supplementary Figs 2 and 3). Interestingly, these compounds were able to be more efficacious and selective than commonly used chemotherapeutics currently in use (e.g. doxorubicin and paclitaxel). An increased efficacy *in vitro* could be in part due to an improved chemical stability that Compound A may possess in comparison to curcumin, which readily degrades as time progresses (Supplementary Fig. 15), or a more efficient binding to the biochemical target(s) in cancerous cells.

The pleiotropic action of curcumin has been well documented to activate apoptosis independent and depend on the cleavage of caspases^36–39^. Apoptotic induction can be a result of a combination of increased ROS, mitochondria membrane potential collapse, and activation of caspases. We have demonstrated that Compound A and I resulted in cancer cell mitochondrial membrane potential collapse within 24 hours of treatment but not in non-cancerous cells (Fig. 4a–e). The mitochondrial membrane potential collapse was further evident by the release of Cyto c as seen in Fig. 4f.

The anti-apoptotic protein BCL-2 is commonly found overexpressed in many human cancers leading to the resistance to common chemo-and radio-therapies used today^40–42^. These analogs were able to decrease BCL-2 expression (Fig. 4g) and maintained their cytotoxicity regardless of BCL-2 status in cancerous cells (Fig. 5a and Supplementary Fig. 4). This mitochondrial membrane potential collapse could be caused by direct interaction of Compound A and I to the mitochondrial proteins leading to destabilization or could be indirectly induced by oxidative stress caused by Compounds A and I. When further chronological analysis of mitochondria membrane potential collapse and ROS production was carried out, we observed that there was early production of oxidative stress within 3 hours of treatment (Fig. 6a–c), whereas mitochondrial destabilization was observed after 6 hours of treatment (*data not shown*). Thus, it seems likely that these compounds target redox systems in cancer cells.

Alterations in the cellular redox environment can lead to the initiation and propagation of a cancerous phenotype^43, 44^. As a result of an increased oxidative stressed environment, cancer cells for their survival, often up-regulate many antioxidant systems to cope with these stresses, relative to their normal counterparts^45^. Thus it is reasonable to suggest the use of molecules to take advantage of such elevated levels for cancer treatment. Natural flavonoids and polyphenols have traditionally been thought of as anti-oxidants, although there is literature to support their use as pro-oxidant compounds *in vitro* and *in vivo*. Such effects are believed to be a result of chelating with transition metal ions, inhibiting mitochondrial respiration, and oxidation of peroxidases or antioxidant molecules^12, 46–50^.

Traditionally, the polyphenol, curcumin has been shown to be a potent anti-inflammatory and antioxidant, but herein we observe a pro-oxidant effect of curcumin and Compounds A and I in cancer cells. If the increased production of ROS is critical for the induction of apoptosis for Compounds A and I, then antioxidants or ROS-scavengers like NAC can rescue the cancer cells. Indeed, cells co-treated with these compounds and NAC showed drastically reduced ROS generation (Fig. 6a–c) and near complete reduction of apoptotic markers (Fig. 6d–f and Supplementary Fig. 7) as well as caspase cleavage or γH2AX (Fig. 6g). Furthermore, cells treated with Compound A and rescued by NAC were able to resume normal cell proliferation when plated in fresh media, whereas Compound A treated cells without NAC could not resume a similar proliferative capability (Fig. 6h). These results indicate that ROS production caused by these compounds is the initial and critical step towards induction of apoptosis in cancer cells. More importantly, these compounds did not induce appreciable ROS production in non-cancerous cells (Fig. 6j) indicating their potential selectivity to cancer cells.

These results were complimented by gene expression analysis using RT^2^ PCR that demonstrated differential gene expression following Compound A treatment in cancer cells compared to their normal counterpart (Fig. 7 and Supplementary 10 and Tables 2–8).

In general, it appears that Compound A was able to selectively decrease the expression of several genes in the colorectal cancer cell line responsible for the defenses against ROS including but not limited to several proteins involved in glutathione-homeostasis including peroxiredoxins, glutathione peroxidases, and glutathione S-transferases. Our results indicated that cancer cells are relatively vulnerable to induction of oxidative stress compared to normal cells. Indeed, this hypothesis has also been supported by other researchers who have shown small molecules including piperlongumine^31, 51^, erastin or RSL3^52^, and beta–phenylethyl isothiocyanate^53^ to induce cell death in cancer cells by targeting redox defense systems.

We also observed similar expression profile for a number of genes in both cancer and normal cells following Compound A treatment. The role of these genes may not be critical in the induction of cell death. On the other hand, cancer cells are believed to have higher absolute levels of ROS and antioxidant defenses to ensure their survival. Such differences could potentially render these cells to be more sensitive to absolute changes in these systems either leading to cytostatic or cell death events^5, 9, 54–57^. These results further demonstrate the use of small molecules that potentially could target redox systems differentially in cancerous cells.

Targeting oxidative stress vulnerability of cancerous cells has been shown by the natural compound, PL *in vitro* and *in vivo* ^31^. It plausible that the addition of curcumin analogs to PL could have enhanced effects in killing of cancer cells as we have shown these compounds to induce apoptosis in cancer cells via increased oxidative stress. We observed a synergistic apoptotic effect with the combination of PL and Compound A in different blood and triple-negative breast cancer cell lines with very limited effects on normal cells (Fig. 8c–i, Supplementary Fig. 12). Thus, we present a novel combination strategy, which both hinders the cellular oxidative stress defense mechanisms and induces ROS generation to achieve an enhanced anti-cancer response. Furthermore, we have also observed that Compound A in combination with a common cocktail of chemotherapeutics, FOLFOX (composed of Folinic acid, Fluorouracil, and Oxaliplatin) had an additive effect at inducing apoptosis and mitochondria collapse in the colorectal cancer cell line, HCT-116 (Supplementary Fig. 15).

The *in vivo* anti-cancer efficacy of natural curcumin and previously reported analogs have been less than satisfactory in terms of inhibiting human tumor xenograft growth or with the administration of high amounts of compound^58–62^. Interestingly, our results showed that intraperitoneally administered Compound A was very effective in reducing the volume of both human leukemia and triple-negative cancer xenograft in mice at 5.0 and 7.5 mg/kg respectively (Fig. 9a,b). The fact that intraperitoneal administration was efficient in reducing subcutaneous xenograft growth suggests that Compound A is absorbed sufficiently and is stable in physiological conditions. Immunohistochemical analysis shows Compound A to increase levels of cleaved caspase-3 and decrease the levels of the proliferation marker, PCNA compared to the control tumors (Fig. 9c), suggesting that this treatment is able to effectively induce apoptosis and reduce cell proliferation in the tumor tissue.

Notably, administration of Compound A in mice for up to 5 weeks did not cause significant changes in weight compared to control mice, thus, indicating that Compound A is well tolerated. Immunohistochemistry further supports the tolerability of Compound A as no differences were observed between the amounts of γH2AX in the kidney tissues from control and Compound A treated animals (Supplementary Fig. 14). Further work will be required to evaluate whether or not Compound A has a greater bioavailability compared to natural curcumin.

In summary, the novel MAC, Compound A has great potential for further development for safe and effective treatment for human cancers. Compound A is able to exert anti-cancer effects not only *in vitro* but effectively *in vivo* when administered in the peritoneal cavity. It is well tolerated, and selective in inducing apoptosis in cancerous cells by the production of ROS. This activity and selectivity may be attributed to the differential redox states that may exist between cancerous and non-cancerous cells. A limitation of this work is the lack of direct target(s) that Compound A may be affecting, whether it is in fact a protein belonging to one of these redox defense systems or pathways upstream of them and will require future investigations to fully elucidate the compounds activity. As a whole, these results merit potential avenues of research for the use of Compound A as a mono- or combinatorial therapy by targeting redox systems in cancer cells.

## Materials and Methods

### Chemicals

Cells were treated with curcumin analogs, curcumin (CUR; Sigma-Aldrich Canada, Cat. No. 8511, Mississauga, ON, Canada), taxol (TAX; Sigma-Aldrich Canada, Cat. No. T7402, Mississauga, ON, Canada), staurosporine (STS; Sigma-Aldrich Canada, Cat. No. S4400, Mississauga, ON, Canada), doxorubicin (DOX; Sigma-Aldrich Canada, Cat. No. D1515, Mississauga, ON, Canada), gemcitabine (GEM; Sigma-Aldrich Canada, Cat. No. G6423, Mississauga, ON, Canada), piperlongumine (PL; INDOFINE Chemical Company, Inc., Cat. No. P-004, Hillsborough, NJ, USA), the broad spectrum caspase inhibitor, Z-VAD-FMK (EMD Chemicals, Gibbstown, NJ, USA), antimycin A (AMA; Sigma-Aldrich Canada, Cat. No. A8674, Mississauga, ON, Canada), thenoyltrifluoroacetone (TTFA; Sigma-Aldrich Canada, Cat. No. T27006, Mississauga, ON, Canada), rotenone (ROT; Sigma-Aldrich Canada, Cat. No. R8875, Mississauga, ON, Canada) were all dissolved in dimethylsulfoxide (DMSO) stock solutions. Recombinant human Fas-Ligand (FasL; Sigma-Aldrich Canada, Cat. No. SRP3036, Mississauga, ON, Canada), water soluble Ubisol-Coenzyme Q_10_ (Ubs-CoQ_10_) and its carrier molecule polyoxyethanyl α-tocopheryl sebacate (PTS) were obtained from Zymes LLC, Hasbrouck Heights, NJ, USA. N-Acetyl-L-Cysteine (NAC; Sigma-Aldrich Canada, Cat. No. A7250, Mississauga, ON, Canada) and paraquat (PQ; Sigma-Aldrich Canada, Cat. No.856177, Mississauga, ON, Canada) were prepared in double distilled water stock solutions.

### Chemical Synthesis of Curcumin Analogs

All chemical reagents were obtained from Sigma–Aldrich, Fluka, and Aladdin (Beijing, China). Silica gel (GF254) for thin-layer chromatography and column chromatography (100–200 and 200–300 mesh) were obtained from Aladdin. Most of compounds were synthesized by aldol condensation between aryl aldehydes and ketones (piperid-4-one, 1-methylpiperid-4-one, pyrone or 1-(2-chlorophenyl)ethanone) (Compounds **A-E, G, I and J**) or an additional acylation reaction (Compounds **F** and **H**). The synthesis of these **unreported** compounds is briefly described as following two sections.

### General procedure for synthesis of compound **A**-**E**, **G** and **I**

The piperid-4-one hydrochloride (2 mmol) and corresponding aryl (4 mmol) aldehydes were dissolved in the mixture solvent of ethanol and water (10:1) to prepare Compound **B**, **C**, **G**, and **I**, while 1-methylpiperid-4-one or pyrone (2 mmol) and corresponding aryl aldehydes (4 mmol) were dissolved in absolute ethanol as well as 1-(2-chlorophenyl)ethanone (4 mmol) and 1,4-Phthalaldehyde (2 mmol). The Compounds **D** containing –OH group in the benzene ring were catalyzed by HCl gas. Other compounds were catalyzed by 40%NaOH at 5–8 °C. All reactions were monitored by the silica gel TLC. At the end of the reaction, water is added into the reaction mixture to precipitate the product. The crude were purified by column chromatography using PE/EA or CH_2_Cl_2_/CH_3_OH.

### Preparation of compound **F** and **H**

A mixture of piperid-4-one hydrochloride (3 mmol) and 3,4,5-trimethoxybenzaldehyde (6 mmol) in ethanol and water (10:1) was stirred at 5–8 °C. 40% NaOH solution (0.5 mL) was added in. After being stirred overnight, the resulting mixture was poured into cold water (10 mL) to precipitate the product. The filter residue was dried in the oven at 40 °C and used for next step reaction without further purification. A solution of isobutyryl chloride or acetyl chloride (2 mmol) in dichloromethane (2 mL) was added dropwise into a mixture of dried residue (1 mmol) and triethylamine (0.25 mL) in dichloromethane (8 mL) under the condition of ice-water bath (5–8 °C). After being stirred overnight at room temperature, the solvent was removed under vacuum, and extracted with dichloromethane twice and dried over MgSO_4_. The organic layer was concentrated in vacuum and refined by chromatography to give the Compound **F** and **H**.

### Stability Assessment Assay

Each compound was dissolved in 1 mL DMSO and diluted by DMSO to a final concentration of 1 mM/L, then 2 μL solution and 98 μL phosphate buffer (pH 7.4) were mixed in the eppendorf tube. The optical density values from 250 to 600 nm were determined using a Spectrum Max M5 (Molecular Devices, USA). Taking 5 minutes as intervals, the absorption curve was recorded for over 25 minutes. The measured setting was at 25 °C at varying time interval in a 1 cm path-length quartz cuvette. In this experiments, the decline of absorption spectrum at different moments was interpreted as the degradation of compound, which reflects the stability of compound *in vitro*.

### Cell Culture

The E6-1 acute T-cell leukemia cell line (American Type Culture Collection, Cat. No. TIB-152, Manassas, VA, USA), was cultured with RPMI-1640 medium (Sigma-Aldrich Canada, Mississauga, ON, Canada) supplemented with 10% (v/v) fetal bovine serum (FBS) standard (Thermo Scientific, Waltham, MA, USA) and 10 mg/mL gentamicin (Gibco BRL, VWR, Mississauga, ON, Canada).

The dominant-negative Fas-Associated protein with Death Domain (FADD) Jurkat cell line (dnFADD Jurkat: ATCC, Cat. No.CRL-2572, Manassas, VA, USA) was cultured with RPMI-1640 medium (Sigma-Aldrich Canada, Mississauga, ON, Canada) supplemented with 10% (v/v) fetal bovine serum (FBS) standard (Thermo Scientific, Waltham, MA, USA) and 10 mg/mL gentamicin (Gibco BRL, VWR, Mississauga, ON, Canada).

The BCL-2 Jurkat cell line (ATCC, Cat. No.CRL-2899, Manassas, VA, USA) was cultured with RPMI-1640 medium (Sigma-Aldrich Canada, Mississauga, ON, Canada) supplemented with 10% (v/v) fetal bovine serum (FBS) standard (Thermo Scientific, Waltham, MA, USA) and 10 mg/mL gentamicin (Gibco BRL, VWR, Mississauga, ON, Canada).

The acute monocytic leukemia cell line, THP-1 (ATCC, Cat. No. TIB-202, Manassas, VA, USA), was cultured with RPMI-1640 medium (Sigma-Aldrich Canada, Mississauga, ON, Canada) supplemented with 0.05 mM beta-mercaptoethanol, 10% (v/v) fetal bovine serum (FBS) standard (Thermo Scientific, Waltham, MA, USA) and 10 mg/mL gentamicin (Gibco BRL, VWR, Mississauga, ON, Canada).

The MV-4-11, chronic myelomonocitic leukemia cell line (ATCC, Cat. No. CRL-9591, Manassas, VA, USA), was cultured with Iscove’s Modified Dulbecco’s Medium (ATCC, Cat. No. 30–2005, Manassas, VA, USA) supplemented with 10% (v/v) FBS standard (Thermo Scientific, Waltham, MA, USA) and 10 mg/mL gentamicin (Gibco BRL, VWR, Mississauga, ON, Canada).

The U-937 histiocytic lymphoma cell line (ATCC, Cat. No. CRL-1593.2, Manassas, VA, USA), was cultured with RPMI-1640 medium (Sigma-Aldrich Canada, Mississauga, ON, Canada) supplemented with 10% (v/v) fetal bovine serum (FBS) standard (Thermo Scientific, Waltham, MA, USA) and 10 mg/mL gentamicin (Gibco BRL, VWR, Mississauga, ON, Canada).

The A549 non-small cell lung cancer cell line (ATCC, Cat. No. CRM-CCL-185, Manassas, VA, USA) was grown and cultured in F-12K medium (ATCC, Cat. No. 30–2004, Manassas, VA, USA), supplemented with 10% (v/v) FBS standard (Thermo Scientific, Waltham, MA, USA) and 10 mg/mL gentamicin (Gibco BRL, VWR, Mississauga, ON, Canada).

The NCI-H23 non-small cell lung cancer cell line (ATCC, Cat. No. CRL-5800, Manassas, VA, USA), was grown and cultured in RPMI-1640 medium (Sigma-Aldrich Canada, Mississauga, ON, Canada) supplemented with 10% (v/v) FBS standard (Thermo Scientific, Waltham, MA, USA) and 10 mg/mL gentamicin (Gibco BRL, VWR, Mississauga, ON, Canada).

The MDA-MB-231 and MDA-MB-468 triple-negative breast adenocarcinoma cell lines (ATCC, Cat. No. HTB-26 & HTB-132, Manassas, VA, USA) were cultured with Dulbecco’s Modified Eagles Medium HAM F12 (Sigma-Aldrich, Mississauga, ON, Canada) supplemented with 10% (v/v) FBS standard (Thermo Scientific, Waltham, MA, USA) and 10 mg/mL gentamicin (Gibco BRL, VWR, Mississauga, ON, Canada).

The SUM149, inflammatory, triple-negative breast cancer cell line (a generous gift from Dr. Stephen Ethier, Wayne State University, Detroit, MI, USA) was cultured in Dulbecco’s Modified Eagles Medium HAM F12 (Sigma-Aldrich, Mississauga, ON, Canada) supplemented with 5% (v/v) FBS standard (Thermo Scientific, Waltham, MA, USA), 10 mg/mL gentamicin (Gibco BRL, VWR, Mississauga, ON, Canada), 5 μg/ml insulin (Sigma-Aldrich, Mississauga, ON, Canada), and 1 μg/ml hydrocortisone (Sigma-Aldrich, Mississauga, ON, Canada).

A pancreatic adenocarcinoma cell line, BxPC-3 (ATCC, Cat. No. CRL-1687, Manassas, VA, USA), was cultured in RPMI-1640 medium (Sigma-Aldrich Canada, Mississauga, ON, Canada) supplemented with 10% (v/v) fetal bovine serum (FBS) standard (Thermo Scientific, Waltham, MA, USA) and 10 mg/mL gentamicin (Gibco BRL, VWR, Mississauga, ON, Canada).

The human colorectal cancer cell line HCT-116 (ATCC, Cat. No. CCL-247, Manassas, VA, USA) was cultured with McCoy’s Medium 5a (Gibco BRL, VWR, Mississauga, ON, Canada) supplemented with 2 mM L-glutamine, 10% (v/v) FBS (Thermo Scientific, Waltham, MA, USA) and 10 mg/ml gentamicin (Gibco, BRL, VWR, Mississauga, ON, Canada).

The osteosarcoma cell lines, MG-63 and Saos-2 (ATCC, Cat. No. CRL-1427 & HTB-85, Manassas, VA, USA), were grown in McCoy’s 5A Medium Modified (Sigma-Aldrich Canada, Mississauga, ON, Canada). The MG-63 medium was supplemented with 10% (v/v) FBS standard (Thermo Scientific, Waltham, MA, USA) and 10 mg/mL gentamicin (Gibco BRL, VWR, Mississauga, ON, Canada). The Saos-2 medium was supplemented with 15% (v/v) FBS standard (Thermo Scientific, Waltham, MA, USA) and 10 mg/mL gentamicin (Gibco BRL, VWR, Mississauga, ON, Canada).

The normal-derived colon mucosa (NCM460) cell line (INCELL Corporation, LLC, San Antonio, TX, USA) was grown in RPMI-1640 medium (Sigma-Aldrich Canada, Mississauga, ON, Canada) supplemented with 10% (v/v) FBS and 10 mg/mL gentamicin (Gibco BRL, VWR, Mississauga, ON, Canada).

The normal human fibroblast cell line, AG09309 (NHF; Coriell institute for Medical Research, Cat. No. AG09309, Camden, New Jersey, New York, USA) was cultured with Eagle’s Minimum Essential Medium (Sigma-Aldrich Canada, Mississauga, ON, Canada) supplemented with 15% (v/v) FBS standard (Thermo Scientific, Waltham, MA, USA), 10 mg/mL gentamicin (Gibco BRL, VWR, Mississauga, ON, Canada) and 5% MEM non- essential amino acids solution (100x) (Thermo Scientific, Cat. No. 11140–050, Waltham, MA, USA). The non-tumorigenic epithelial breast cell line, MCF10A (ATCC, Cat. No. CRL-10317, Manassas, VA, USA) was cultured in Mammary Epithelial Cell Growth Medium (MEGM; Lonza/Clonetics Corporation, Cat. No. CC-3150, Walkersville, MD, USA) with the MEGM Growth Kit (Lonza/Clonetics Corporation, Cat. No. CC-3150, Walkersville, MD, USA) with slight modifications: 10 mg/mL of gentamicin and 160 ng/mL of cholera toxin.

All cells were grown in optimal growth conditions of 37 °C and 5% CO_2_. Furthermore, all cells were cultured and passaged for less than 3 months and the authors performed no authentication of cell lines.

### Peripheral Blood Mononuclear Cell Isolation

Peripheral blood mononuclear cells (PBMCs) were collected and isolated from healthy volunteers with informed consent from each subject and were done with prior approval following the relevant guidelines and regulations of the Research Ethics Board of the University of Windsor (protocol # REB # 04–147). In brief, whole blood was collected in BD Vacutainer CPT Tubes with Sodium Heparin (Becton, Dickinson and Company, Cat. No. 362753, Franklin Lakes, NJ, USA) at room temperature. Tubes were immediately inverted 5 times and centrifuged for 30 minutes at room temperature at 1500–1800 × g. The layer of PBMCs under the plasma layer in each tube was collected, pooled together, resuspended in 50 mL of PBS, and centrifuged at room temperature at 300 × g for 15 minutes. The supernatant was aspirated without disturbing the pellet and PBMCs were resuspended and cultured in RPMI-1640 medium (Sigma- Aldrich Canada, Mississauga, ON, Canada), supplemented with 10% (v/v) FBS standard (Thermo Scientific, Waltham, MA, USA) and 10 mg/mL gentamicin (Gibco BRL, VWR, Mississauga, ON, Canada) at 37 °C and at 5% CO_2_.

### WST-1 Assay for Cell Viability

The water-soluble tetrazolium salt (WST-1) is a colorimetric based assay to quantify cell viability through cellular metabolism. Assay was performed similar to the manufacturer’s protocol (Roche Applied Science, Indianapolis, IN, USA). 96-well clear bottom tissue culture plates were used and seeded with the following cell concentrations: E6-1, MV-4-11, and U-937 at 5 × 10^3^ cells/well, A549 and NCI-H23 at 3 × 10^3^ cells/well, Saos-2 and MG-63 at 6 × 10^3^ cells/well, MDA-MB-231 and −468 at 4 × 10^3^ cells/well, HCT-116 at 2 × 10^3^ cells/well, and BxPC-3 at 3 × 10^3^ cells/well. Following seeding, cells were incubated over night followed by treatment with the indicated concentration of the indicated compound for 48 hours. WST-1 reagent was added for 4 hours at 37 °C and monitored for formazan production by measuring absorbance at 450 nm on a Wallac Victor 1420 Multilabel Counter (PerkinElmer, Woodbridge, ON, Canada). Results are expressed as percentage to the solvent control group (DMSO). Inhibitory dose-response curves (log(inhibitor) vs. response – Variable slope (four parameters)) were calculated using GraphPad Prism 6.

### Apoptotic Detection by Annexing V binding Assay and Propidium Iodide (PI) Staining

Cells were seeded in 6-well plates at 275,000 cells/well if suspension or 100,000 cells/well if adherent and allowed to incubate overnight. Cells were treated with vehicle control (DMSO) or indicated compound for a specified amount of time. Cells were collected and spun down at 3000 rpm, washed in 1xPBS and resuspended in Annexin V binding buffer (10 mM HEPES, 140 mM NaCl, 2.5 mM CaCl_2_, pH 7.4), incubated for 15 minutes at 37 °C with the Annexin V Alexa-Fluor-488 (1:20) (Life Technologies Inc, Cat. No. A13201, Burlington, On, Canada) and propidium iodide (PI; 0.01 mg/mL) (Life Technologies Inc, Cat. No. P3566, Burlington, On, Canada).

For quantification, the Tali Image-Based Cytometer. (LifeTechnologies Inc, Cat. No. T10796, Burlington, ON, Canada). Each group had 20 random fields analyzed using green (excitation 458 nm; emission 525/20 nm) and red (excitation 530 nm; emission 585 nm) channels. Results were expressed as percentage of cells for early (green), late apoptotic cells (green and red), and necrotic cells (red). Viable cells were considered as those that were not positive for both Annexin V and PI.

For qualitative measurement of apoptosis, a similar procedure was carried out with the exception of adherent cells being grown and imaged on 35 mm Glass Bottom Culture Dishes (MatTek Corporation, Cat. No. P35G-0.170-14-C, Ashland, MA, USA) and suspension cells were imaged on a microscope slide with cover slips. Furthermore, cells were counterstained with Hoechst 33258 (Sigma-Aldrich Canada, Cat. No. 861405, Mississauga, ON, Canada) with a final concentration of 10 μM during the 15 minute incubation. All groups were subjected to micrographs that were taken on a Leica DM IRB inverted fluorescence microscope (Leica Microsystems, Wetzlar, Germany) at 20x, or 40x objective lens.

### Mitochondrial Membrane Potential Measurement

Tetramethylrhodamine methyl ester (TMRM) was used to monitor mitochondria membrane potential (MMP). Cells were seeded in 6-well plates at 275,000 cells/well if suspension or 100,000 cells/well if adherent and allowed to incubate overnight. Cells were treated with vehicle control (DMSO) or indicated compound for a specified amount of time. Cells were collected and allowed to incubate in TMRM at a final concentration of 100 nM for 45 minutes.

For quantification, cells were collected and washed in 1xPBS before subjected to image-based cytometry using the Tali Image-Based Cytometer. (LifeTechnologies Inc, Cat. No. T10796, Burlington, ON, Canada). Results were quantified as percentage of cells positive for TMRM.

Fluorescent micrographs were carried out in similar procedure with the exception of adherent cells being grown and imaged on 35 mm Glass Bottom Culture Dishes (MatTek Corporation, Cat. No. P35G-0.170-14-C, Ashland, MA, USA) and suspension cells were imaged on a microscope slide with cover slips. Furthermore, cells were counterstained with Hoechst 33258 (Sigma-Aldrich Canada, Cat. No. 861405, Mississauga, ON, Canada) at a final concentration of 10 μM during the 45 minute incubation. Micrographs were taken on a Leica DM IRB inverted fluorescence microscope (Leica Microsystems, Wetzlar, Germany) at 20x, or 40x objective lens’.

### Whole Cell Detection of Reactive Oxygen Species

7′-dichlorofluorescin diacetate (DCFDA) was used to detect whole cell ROS. Cells were seeded in 6-well plates at 275,000 cells/well if suspension or 100,000 cells/well if adherent and allowed to incubate overnight. DCFDA was pretreated for 30 minutes at a final concentration of 20 μM at 37 °C then cells were treated with the negative control (DMSO vehicle) or the compounds being studied (dissolved in DMSO or water) for 3 or 6 hours protected from light. For the 24-hour time point, cells were treated first with studied compound or DMSO then treated with DCFDA at the 18-hour time point for 6 hours. Cells were then collected in 2 mL eppendorfs followed by centrifugation at 2000 RPM for 5 minutes. Media was aspirated and resuspended in 1xPBS. Results were quantified using the Tali Image-Based Cytometer (LifeTechnologies Inc, Cat. No. T10796, Burlington, ON, Canada) and were measured as the percent of cells positive for the green fluorescent product, DCF relative to the DMSO control.

### Growth Curve Post-Treatment Removal

Cells were plated and treated with their respective doses for a period of time. Cells were then collected and washed in 1xPBS. Each group was re-plated with the same amount of cells with fresh media not containing any treatments and then allowed to recover for 24 or 48 hours. Cells were then collected and the number of healthy cells was counted using trypan blue (Sigma-Aldrich Canada, Cat. No. T8154, Mississauga, ON, Canada). For the E6-1 cell line 100,000 cells were plated to begin the assay, treated for 24 hours and re-plated with 50,000 cells with fresh media for 24 or 48 hours. For the MBA-MD-231 cell line 100,000 cells were plated to begin the assay, treated for 48 hours, then re-plated with 30,000 cells with fresh media for 24, 48, or 72 hours.

### Cell Lysis

Cells were plated in 10 cm petri dishes at 2 × 10^6^ if suspension or 1.5 or 1 × 10^6^ cells/dish if adherent and allowed to incubate overnight. Cells were treated with indicated compounds or DMSO for the designated time point. Cells were then collected and washed once with PBS. Cells were then suspended in ice cold lysis buffer (1% Triton X-100, 50 mM Tris-HCl, 150 mM NaCl, 0.1% SDS and ddH_2_O, pH = 7.3), with HaltTM Protease Inhibitor Cocktail (100X) (ThermoFisher Scientific, Cat. No. 78438, Waltham, MA, USA) using manufactures protocol and incubated on ice for 5 minutes. Using a P1000, slowly pipetting ten times to lyse the cells. Cells were spun down at 10,000 RPM at 4 °C for 10 minutes to separate the cytosolic content and cell debris. Using the Bradford assay, each sample had approximately 30 μg of protein.

### Cytosol-Mitochondria Fraction Isolation

Cells were plated in 10 cm petri dishes at 3 × 10^6^ to incubated overnight. Cells were then treated with vehicle control or compound for 24 hours. Mitochondria and cytosolic fractions of each group were isolated utilizing the Mitochondria Isolation Kit for Culture Cells (Abcam Canada, Cat. No. ab110170, Toronto, ON, Canada) following the manufacture’s protocol. Following separation, a Bradford assay was conducted, cytosolic and mitochondria fractions had approximately 20 μg of protein. Each sample was then subjected to Western blot analysis.

### Western Blot Analysis

SDS-PAGE was performed on the protein samples followed by transfer to a PVDF membrane. Membranes blocked in 5% BSA or skim milk for at least 1 hour. Membranes incubated with the primary antibodies caspase-8 (1:1000, Cell Signaling Technology, Cat No. 9746S, Danvers, MA, USA), and γ-H2AX (Ser139) (1:1000, Santa Cruz Biotechnology, Cat. No. sc-101586, Dallas, TX, USA) were blocked and incubated overnight in 5% BSA. Membranes incubated the primary antibodies caspase-3 (1:2000, Novus Biologicals, Cat. No. NB100-56709, Littleton, CO, USA), caspase-9 (1:1000, Cell Signaling Technology, Cat No. 9502S, Danvers, MA, USA), beta-actin (1:2000, Santa Cruz Biotechnology, Cat. No. sc-47778, Dallas, TX, USA), BCL-2 (1:500, Santa Cruz Biotechnology, Cat. No. sc-7382, Dallas, TX, USA), Bax (1:500, Santa Cruz Biotechnology, Cat. No. sc-7480, Dallas, TX, USA), SDHA (1:1000, Santa Cruz Biotechnology, Cat. No. sc-59687, Dallas, TX, USA), and cytochrome c (1:1000, Abcam Canada, Cat. No. ab13575, Toronto, ON, Canada) were blocked and incubated overnight in 5% skim milk. Following primary antibody incubation, membranes were washed for 10 minutes three times with TBST and were incubated with a goat anti-mouse (1:2000) (Novus Biologicals, Cat. No. NBP2- 30347H, Littleton, CO, USA) or goat anti-rabbit goat anti- rabbit (1:2000) (Novus Biologicals, Cat. No. NBP2-30348H, Littleton, CO, USA) conjugated to horseradish peroxidase-conjugated secondary antibody for 1 hour at 4 °C. After incubation, membranes were washed for five minutes three times with TBST. Bands were visualized with the chemiluminscent SuperSignalTM West Femto Maximum Sensitivity Substrate Solution (ThermoFisher Scientific, Cat. No.34095, Waltham, MA, USA).

### Gene Expression Profiling

Following treatment, HCT-116 and NCM-460 RNA was extracted using the Qiagen RNeasy Mini Kit (Qiagen Inc., Cat. No. 74106, Toronto, ON, Canada). Quality of RNA was determined using the A_280_:A_260_ (Nanodrop 2000). Over 3 μg of RNA was used for each independent trial and the first strand cDNA was produced using the RT^2^ First Strand Kit (Qiagen Inc., Cat. No. 330401, Toronto, ON, Canada). Both protocols were followed as per manufacturer’s protocol with slight modifications. For halting of enzymatic activity for the genomic DNA elimination and the cDNA synthesis, samples were placed at −20 °C until frozen.

Following cDNA synthesis, reverse-transcriptase real-time polymerase chain reaction was performed using the RT^2^ Profiler PCR Array Human Oxidative Stress Plus Array (Qiagen Inc., Cat. No. PAHS-065Y, Toronto, ON, Canada). The array contained 84 different primers related to oxidative stress genes and was performed following the manufacturers protocol utilizing the SYBR Green system. Experimental controls included 5 housekeeping genes, a positive control and a negative control.

Real time amplification data was acquired using the ABI ViiA 7 software. Amplification occurred for 40 cycles for 15 seconds at 95 °C and 1 minute at 60 °C. A melting curve was produced for each sample to confirm specificity of amplification and gene expression was normalized to housekeeping genes. Results are shown as a fold change in gene expression between the controls and treated groups using the ΔΔC_T_ method.

### Xenograft Models

Immunocomprised CD-1 NU male mice (Charles River Laboratories, QC, Canada) were used for the MV-4-11 study and CD-1 NU female mice (Charles River Laboratories, QC, Canada) were used for the MDA-MB-231 study. Mice were housed and the protocols were followed using relevant guidelines and regulations that were approved by the University of Windsor Animal Care Committee (AUPP # 14–15) in accordance with the Canadian Animal Care committee in a laboratory setting with 12-hour light/dark cycles. In the MV-4-11 study, mice were injected subcutaneously on both flanks with 5 million cells. The MDA-MB-231 study, mice were injected subcutaneously on one flank with 2 million cells. Both contained equal parts of cell solution and Corning Matrigel Basement Membrane Matrix (VWR International, Cat. No. 47743–715, Mississauga, ON, Canada) to a final volume of 200 μL. Cell solution was then injected using 23-gauge needles with 1 mL syringes. Once tumors established, volumes were calculated using the modified ellipsoid formula: 1/2 (Length × Width^2^). Both studies had treatments occurring three times a week for five weeks and were injected intraperitoneally at varying doses. MV-4-11 study: Approximately 5.0 mg/kg curcumin, 5.0 mg/kg Compound A or %v/v DMSO in 300 μL PBS. MDA-MB-231 study: 7.5 mg/kg Compound A or %v/v DMSO in 400 μL PBS.

The MV-4-11 results are representative of 5 mice for the control (DMSO) and Compound A treated groups, and 4 mice for the curcumin treated group. Each mouse had a tumor on each flank. The MDA-MB-231 results are representative of 3 mice for the control (DMSO) and 4 mice for the Compound A treated group, each group contained one tumor. Weight was monitored for indication of the ability to tolerate treatment groups.

### Immunohistochemistry

MDA-MB-231 tumors and kidneys were harvested and placed in a 10% formaldehyde solution. Three days prior to sectioning, each organ or tumor was transferred to a 30% sucrose (w/v). All samples were sectioned at 20 µm and subjected to immunohistochemistry using either gamma H2AX (p Ser139) antibody (1:500) raised in rabbit (Novus Biologicals, Cat. No. NB100-384, Littleton, CO, USA), cleaved Caspase 3 antibody (1:1000) raised in rabbit (Novus Biologicals, Cat. No. NB100–56113, Littleton, CO, USA), anti-PCNA [pc10] antibody (1:400) raised in mouse (Abcam, Cat. No. ab29, Cambridge, MA, USA), or anti-vascular endothelial growth factor (VEGF; 1:1000, Abcam Canada, Cat. No. ab1316, Toronto, ON, Canada). Prior to overnight incubation with the primary antibodies at 4 °C, each section was incubated in 0.4% H_2_O_2_ for 2 minutes, followed by DAKO universal blocking solution (purchased from Diagnostics Canada Inc., Mississauga) for 30 minutes and in normal goat serum for primary antibodies raised in rabbit or normal horse serum for primary antibodies raised in mouse for 30 minutes at room temperature (as per instructions on anti-rabbit Vecstatin ABC Kit [Cat. No. PK-6101] or anti-mouse Vecstatin ABC Kit [Cat. No. PK-6102], Vector Laboratories). The sections were washed in Tris buffered saline (TBS) for 5 minutes. Following the overnight incubation, the sections were washed twice and incubated in biotinylated anti-rabbit IgG (Vector Laboratories, anti-rabbit Vecstatin ABC Kit [Cat. No. PK-6101]) or biotinylated anti-mouse IgG (Vector Laboratories, anti-rabbit Vecstatin ABC Kit [Cat. No. PK-6102]) for 75 minutes. The sections were then incubated in avidin biotin complex (ABC reagent) for 45 minutes at room temperature. Following two additional TBS washes, the sections were subjected to the peroxidase substrate 3, 3′ diaminobenzidine (DAB) prepared as per the instructions in the kit (Vector Laboratories, DAB Peroxidase (HRP) Substrate Kit [Cat. No. SK-4100]). Sections were then counterstained with Hematoxylin Solution, Gill No. 1 (Sigma-Aldrich Canada, Cat. No. GHS116, Mississauga, ON, Canada) and washed twice in tap water and once in 1xTBS for 5 minutes. The sections were then dehydrated in anhydrous ethanol and xylene and were cover-slipped using Permount for visualization under a microscope.

### Statistics

All statistics were performed by GraphPad Prism 6 statistical software. A p-value below 0.05 was considered significant. Different statistical analysis was performed depending on the variables in each experiment. For the experiments with single variable measurements, which include quantification of mitochondrial membrane potential, and whole cell ROS, One-Way ANOVA (nonparametric) was conducted and each samples’ mean was compared to the mean of the negative control (DMSO) unless otherwise specified. For experiments that were multi-variable such as the quantification of early and late apoptosis Two-Way ANOVA (nonparametric) was used and each samples’ mean was compared to the mean of the negative control (DMSO) unless otherwise specified.

## Electronic supplementary material

Supplementary Data
