# Supplementary material for: Selective Targeting of Cancer Cells by Oxidative Vulnerabilities with Novel Curcumin Analogs
Source: Sci Rep. 2017 Apr 24;7:1105. doi: 10.1038/s41598-017-01230-4 (PMC5430918; doi:10.1038/s41598-017-01230-4)
Supplement: Supplementary file 1 — Supplementary Data [file 41598_2017_1230_MOESM1_ESM.doc]

Selective Targeting of Cancer Cells by Oxidative Vulnerabilities with

Novel Curcumin Analogs

Author list:

Christopher Pignanelli^1^, Dennis Ma^1^, Megan Noel^1^, Jesse Ropat^1^, Fadi Mansour^1^, Colin Curran^1^, Simon Pupulin^1^, Kristen Larocque^1^, Jianzhang Wu^2^, Guang Liang^2^, Yi Wang^2*^, and Siyaram Pandey^1*^

^1^Department of Chemistry and Biochemistry, University of Windsor,

401 Sunset Avenue, Windsor, Ontario N9B 3P4, Canada

Phone: +519-253-3000, ext. 3701

spandey@uwindsor.ca

^2^Chemical Biology Research Center, School of Pharmaceutical Sciences, Wenzhou Medical University

University Town, Chashan, Wenzhou, Zhejiang, 325035, P.R. China

Phone: 86-577-85773060

yiwang1122@gmail.com

Running Title: Targeting Oxidative Susceptibilities of Cancer Cells by Novel Curcumin Analogs

Key Words:

Cancer, mitochondria, apoptosis, cancer therapy, curcumin, synthetic analogs, piperlongumine, drug discovery, xenograft models, oxidative stress


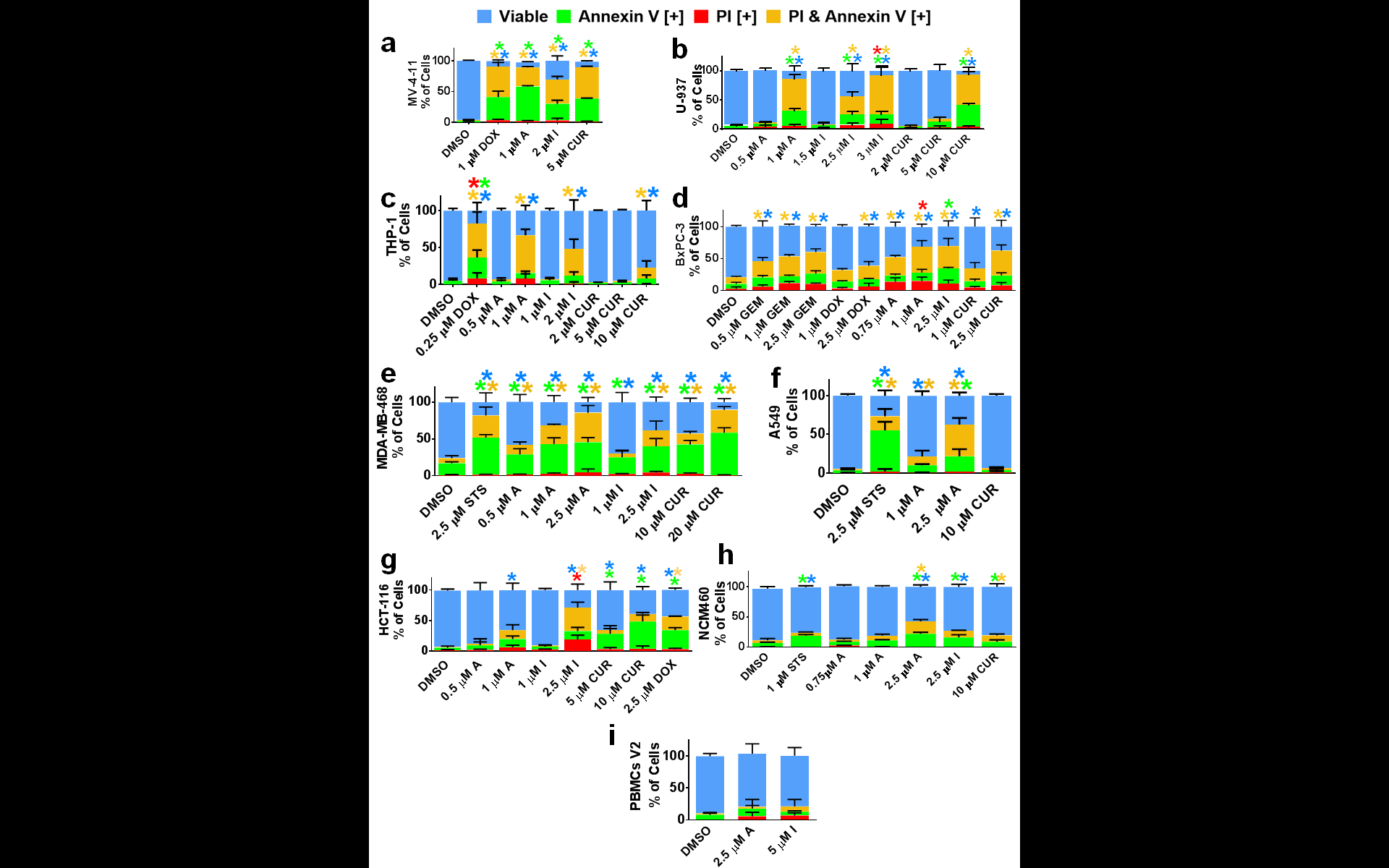


**Supplemental 1. Curcumin Analogs Induce Apoptosis in Several Cancer Cell Lines.** Following treatment with specified doses for 48 hours, cells were stained for Annexin V and PI. Results were obtained using image-based cytometry with the Y-axis representative of percent of cells positive for Annexin V (green), PI (red), Annexin V and PI (yellow), or negative for both Annexin V and PI (blue). Values are expressed as a mean ± SD from three independent experiments. Statistical calculations were performed using Two-Way ANOVA multiple comparison. * p < 0.05 vs Control (DMSO).


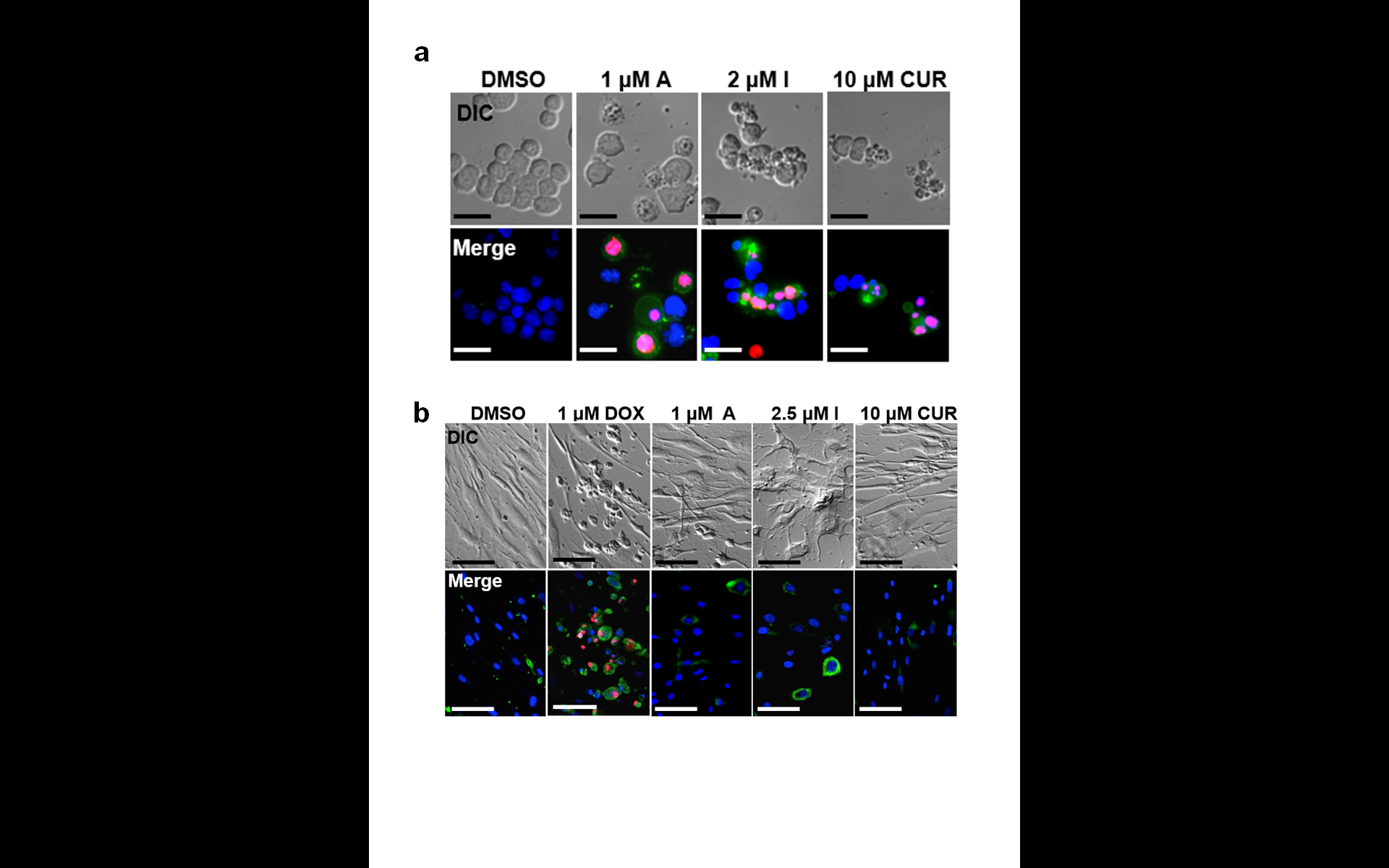


**Supplemental 2. Selective Apoptotic Induction by Curcumin Analogs.** (**a**) E6-1 micrographs at 24 hours following treatment. Top: Bright field DIC images. Bottom: Fluorescent images stained with Annexin V (green), PI (red), and Hoechst (blue). Scale bar is 25 microns. Images are representative of three independent experiments (**b**) NHF micrographs at 48 hours. Top: Bright field DIC images. Bottom: Fluorescent images stained with Annexin V (green), PI (red), and Hoechst (blue). Scale bar is 25 microns. Images are representative of three independent experiments


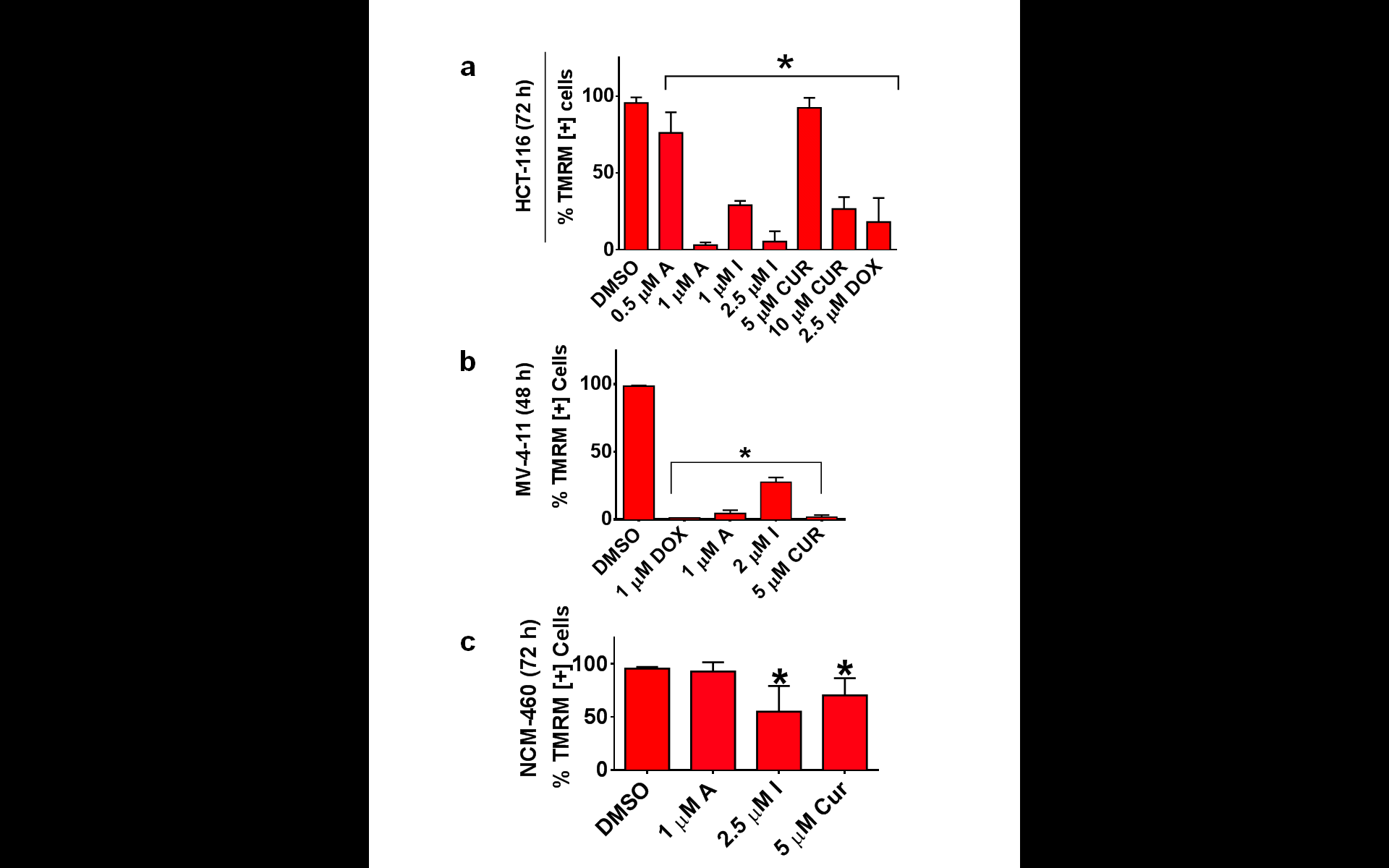


**Supplemental 3. Selective Mitochondria Destabilization Following Treatment with Curcumin Analogs.** (**a-b**) The cancerous cell lines HCT-116, MV-4-11 and normal cell line, NCM-460 were plated and allowed to incubate overnight. Following overnight incubation, cells were treated for 72 or 48 hours. To monitor mitochondria potential cells were incubated with TMRM for 45 minutes before analysis. Results were obtained using image-based cytometry with the Y-axis representative of percent of cells positive for TMRM expressed as a mean ± SD from three independent experiments. Statistical calculations were performed using One-Way ANOVA. * p < 0.05 vs. Control (DMSO).


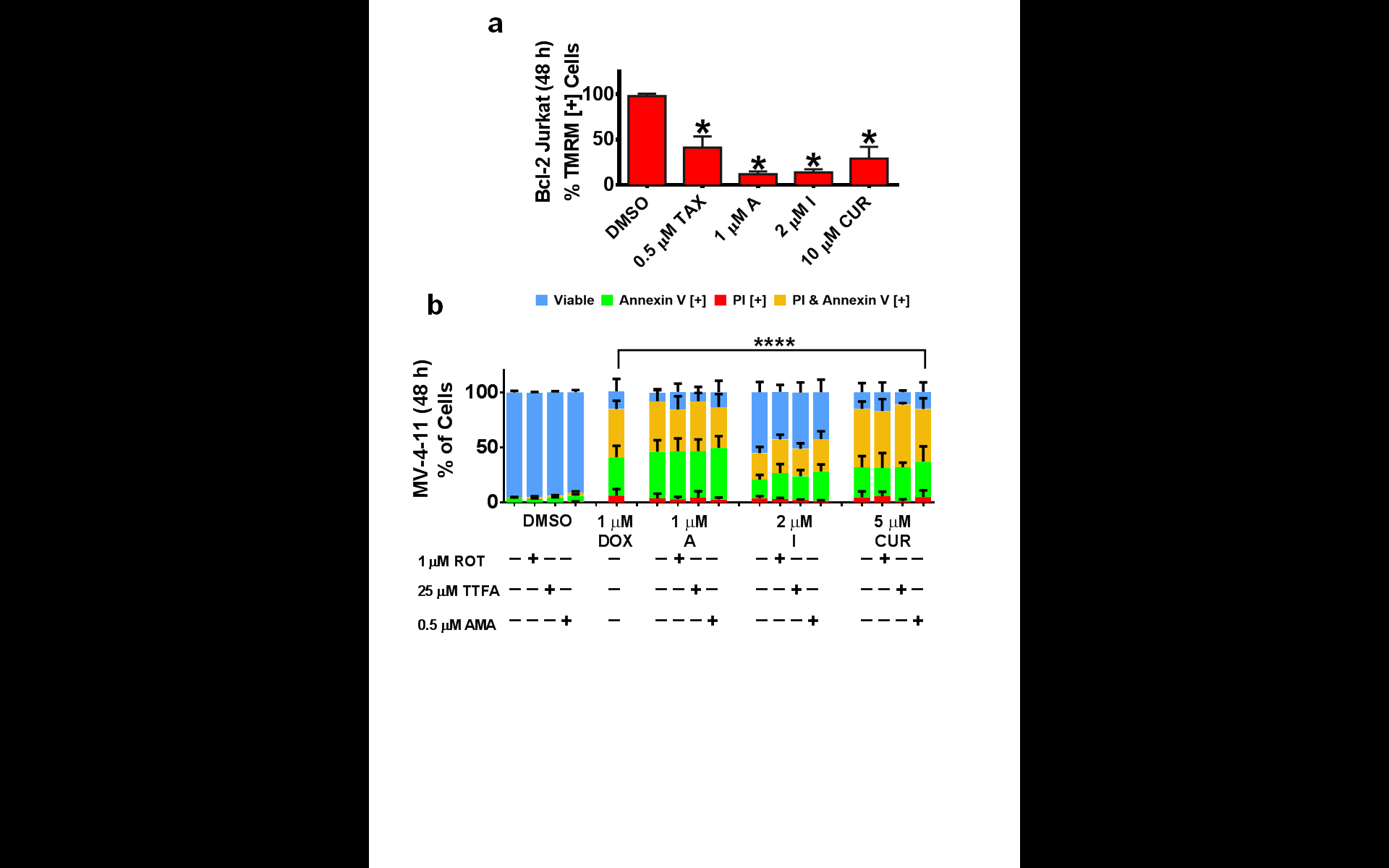


**Supplemental 4. Curcumin Analogs are not Dependent on BCL-2 Status for Mitochondrial Destabilization and Functioning Complex I, II, or III for Apoptotic Induction.** (**a**) Jurkat overexpressing BCL-2 cells were treated for 48 hours. To monitor mitochondria potential cells were incubated with TMRM for 45 minutes before analysis. Results were obtained using image-based cytometry with the Y-axis representative of percent of cells positive for TMRM expressed as a mean ± SD from three independent experiments. (**b**) MV-4-11 cells were pre-treated with either the complex I inhibitor, rotenone (ROT), complex II inhibitor, thenoyltrifluoroacetone (TTFA), or complex III inhibitor, antimycin A (AMA) followed by treatment with curcumin or curcumin analogs for 48 hours. Following treatment, cells were stained for Annexin V and PI. Results were obtained using image-based cytometry with the Y-axis representative of percent of cells positive for Annexin V (green), PI (red), Annexin V and PI (yellow), or negative for both Annexin V and PI (blue). Values are expressed as a mean ± SD from three independent experiments. Statistical calculations were performed using Two-Way ANOVA multiple comparison**.** * p < 0.05 vs % viable cells of Control (DMSO); **** p < 0.01 vs % viable cells of Control (DMSO). Statistical calculations were performed using One-Way ANOVA multiple comparison. * p < 0.05 vs % TMRM [+] cells.


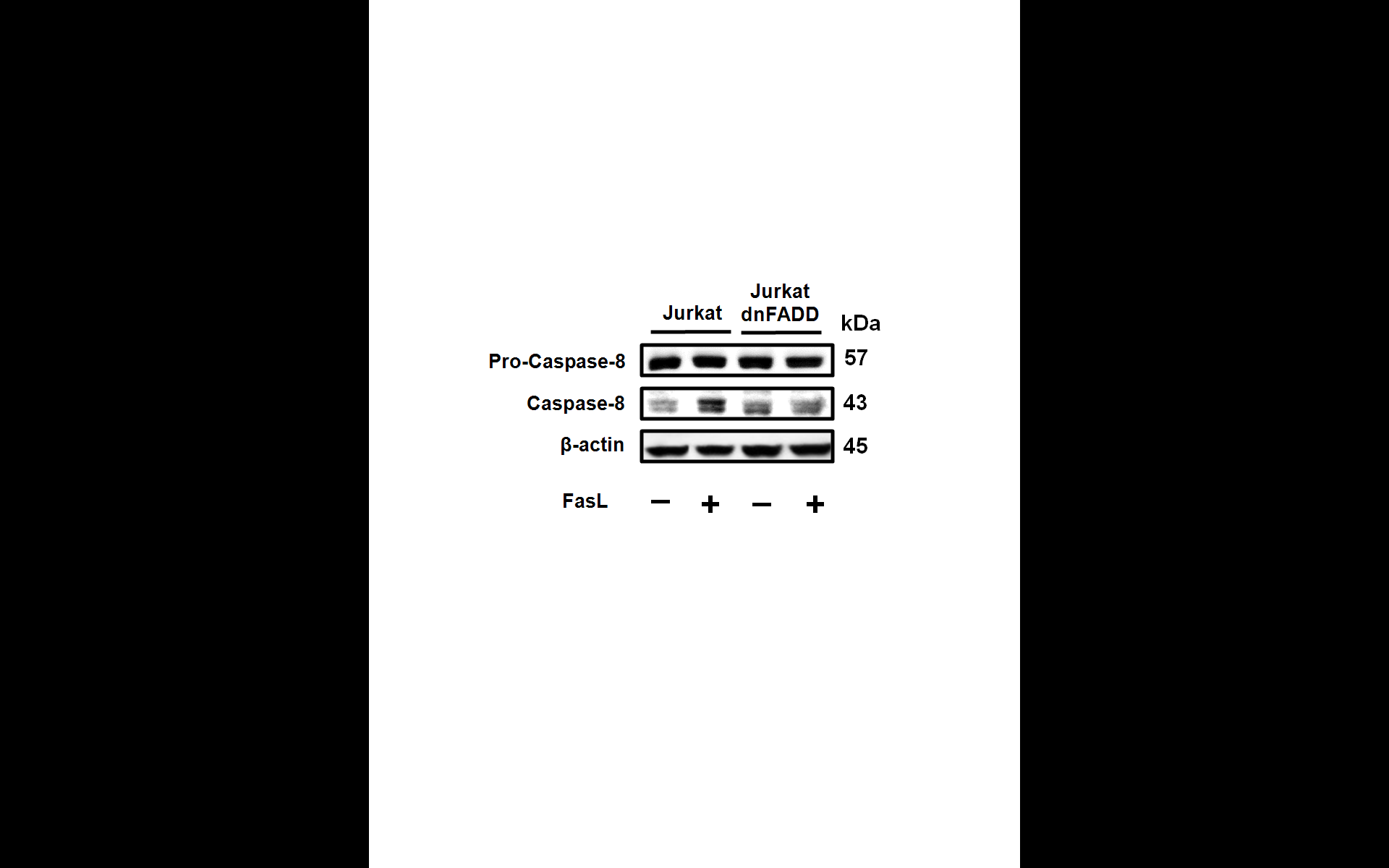


**Supplemental 5. Fas ligand (FasL) Cannot Cause Caspase-8 Cleavage in Cells Lacking Function FADD Protein.** Wild type Jurkat (E6-1) and dnFADD jurkat were treated with or without the FasL for 48 hours. Cells were lysed and protein samples were subjected to SDS-PAGE then transferred to a PVDF membrane. Bands were visualized with a chemiluminescence reagent. Images are representative of three independent experiments.


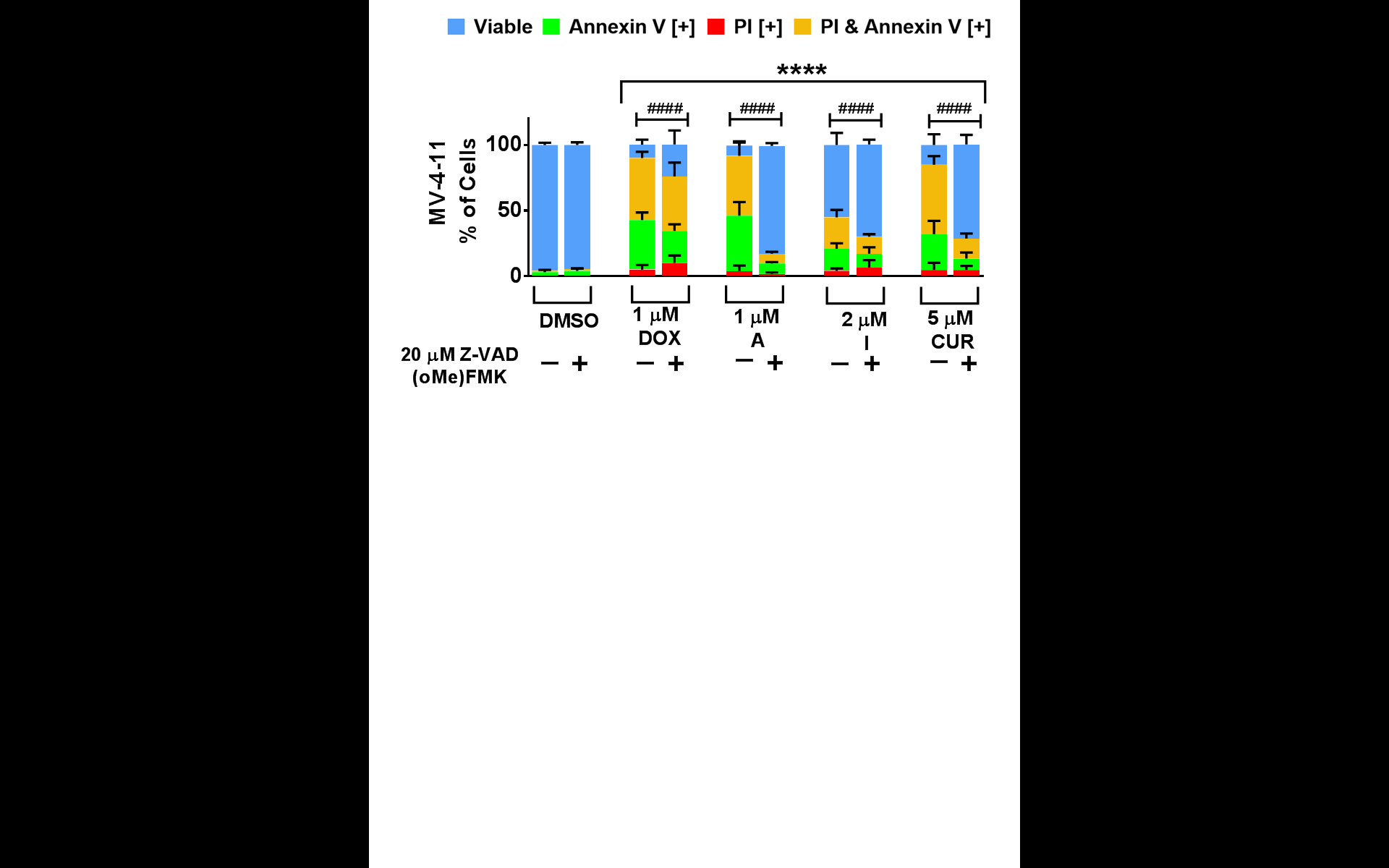


**Supplemental 6. Curcumin Analogs Are Partially Dependent on Caspase Activation for Induction of Apoptosis.** MV-4-11 cells were plated and treated with or without the broad spectrum caspase inhibitor ZVAD(oMe)-FMK for 48 hours. Following treatment, cells were stained for Annexin V and PI. Results were obtained using image-based cytometry with the Y-axis representative of percent of cells positive for Annexin V (green), PI (red), Annexin V and PI (yellow), or negative for both Annexin V and PI (blue). Values are expressed as a mean ± SD from three independent experiments. Statistical calculations were performed using Two-Way ANOVA multiple comparison. **** p < 0.01 vs % viable of Control (DMSO); #### p < 0.01 vs % viable cells for groups without Z-VAD(oMe)FMK.


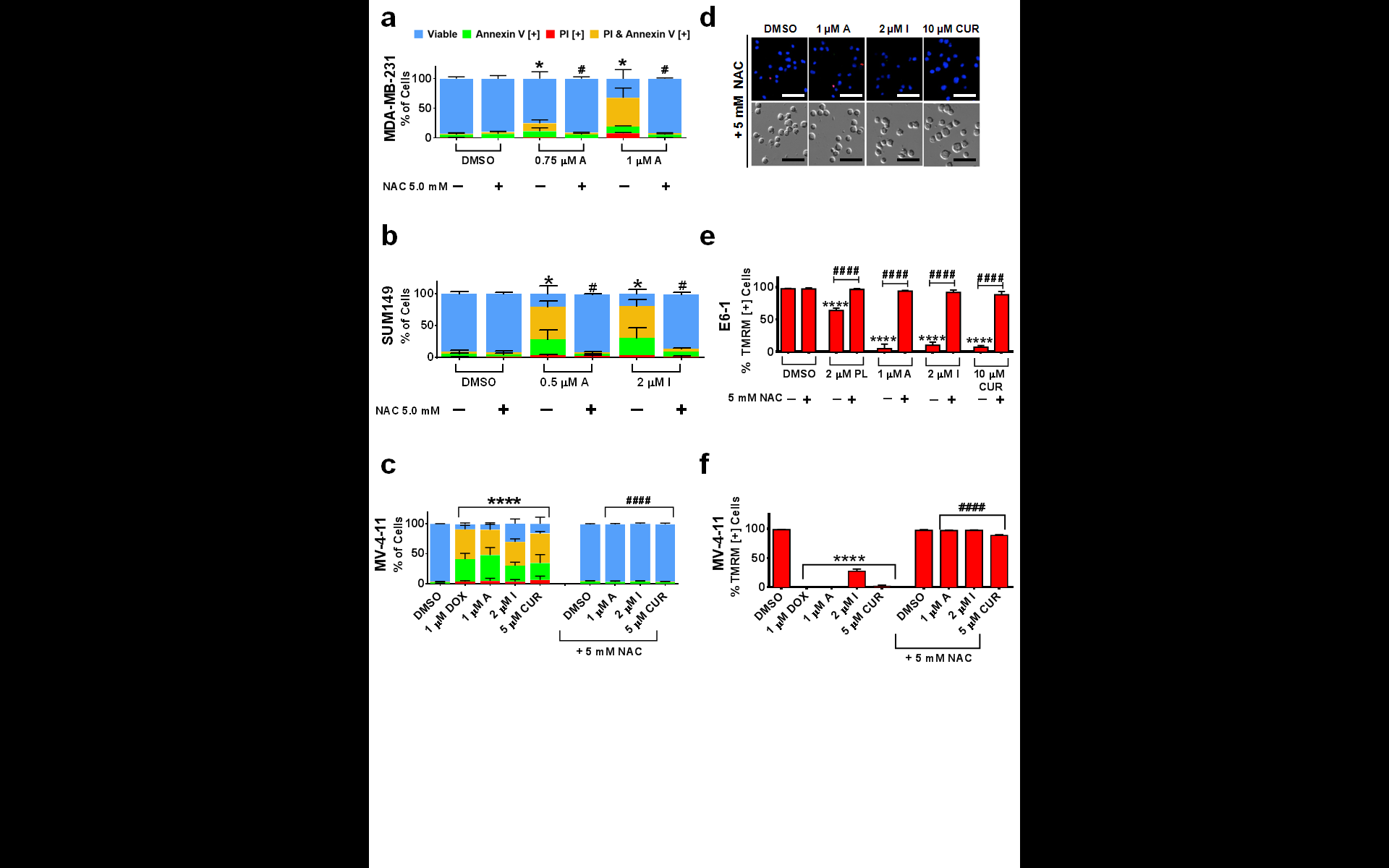


**Supplemental 7. Apoptotic Induction and Mitochondrial Membrane Potential Destabilization of Curcumin Analogs Are Prevented When in Combination with NAC Across a Variety of Cancer Cell Lines.** (**a-c**) Breast cancer (MDA-MB-231, MDA-MB-468 and blood cancer (MV-4-11) cell lines were treated with or without the antioxidant, NAC with curcumin or curcumin analogs for 48 hours. Following treatment, cells were stained for Annexin V and PI. Results were obtained using image-based cytometry with the Y-axis representative of percent of cells positive for Annexin V (green), PI (red), Annexin V and PI (yellow), or negative for both Annexin V and PI (blue). Values are expressed as a mean ± SD from three independent experiments. (**d**) E6-1 micrographs at 48 hours. Top: Fluorescent images stained with Annexin V (green), PI (red), and Hoechst (blue) at 400x magnification. Bottom: Bright field images at 400x magnification. Scale bar is 25 microns. Images are representative of three independent experiments. (**e-f**) E6-1 and MV-4-11 were plated and allowed to incubate overnight. Following overnight incubation, cells were treated for 48 hours with or without NAC. To monitor mitochondria potential cells were incubated with TMRM for 45 minutes before analysis. Results were obtained using image-based cytometry with the Y-axis representative of percent of cells positive for TMRM expressed as a mean ± SD from three independent experiments. Statistical calculations were performed using Two-Way ANOVA multiple comparison**.** * p < 0.05 vs % viable cells of Control (DMSO); **** p < 0.01 vs % viable cells of Control (DMSO) #### p < 0.01 vs % viable cells for groups without NAC. Statistical calculations were performed using One-Way ANOVA multiple comparison. **** p < 0.01 vs % TMRM [+] cells; #### p < 0.01 vs % TMRM [+] cells for groups without NAC.


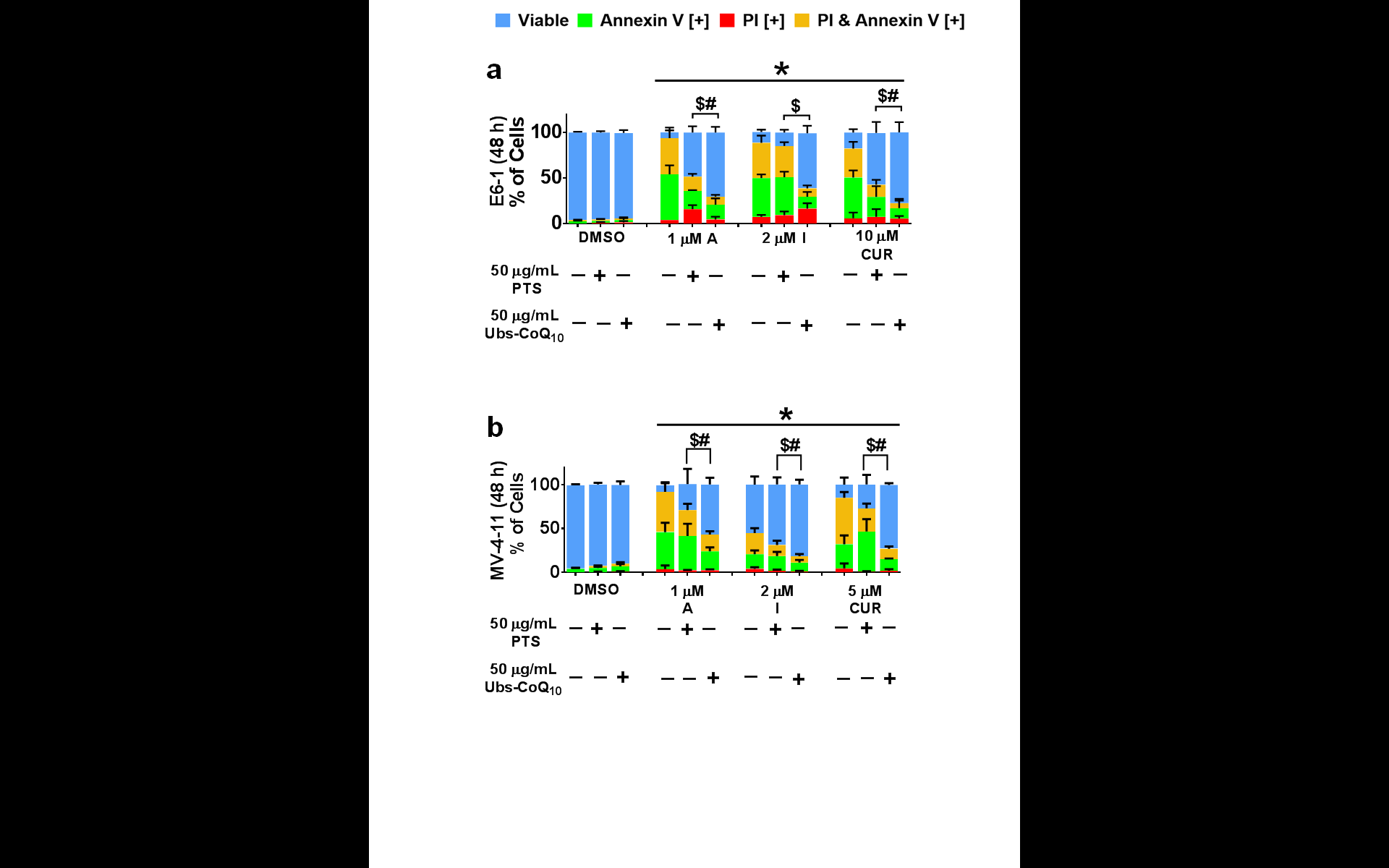


**Supplemental 8. The Water-Soluble Ubisol-Coenzyme Q_10_ is Able to Attenuate Apoptotic Induction of Curcumin Analogs.** E6-1 (**a**) and MV-4-11 (**b**) were treated with or without the antioxidant Ubisol-Coenzyme Q_10_ (Ubs-CoQ_10_), its vehicle control polyoxyethanyl α-tocopheryl sebacate (PTS) along with curcumin and its analogs A and I for 48 hours. Following treatment, cells were stained for Annexin V and PI. Results were obtained using image-based cytometry with the Y-axis representative of percent of cells positive for Annexin V (green), PI (red), Annexin V and PI (yellow), or negative for both Annexin V and PI (blue). Values are expressed as a mean ± SD from three independent experiments. Statistical calculations were performed using Two-Way ANOVA multiple comparison. * p < 0.05 vs % viable cells of Control (DMSO); # p < 0.05 vs % viable cells of groups treated with studied compound alone; $ p < 0.05 vs % viable cells of combination of PTS and studied compound.


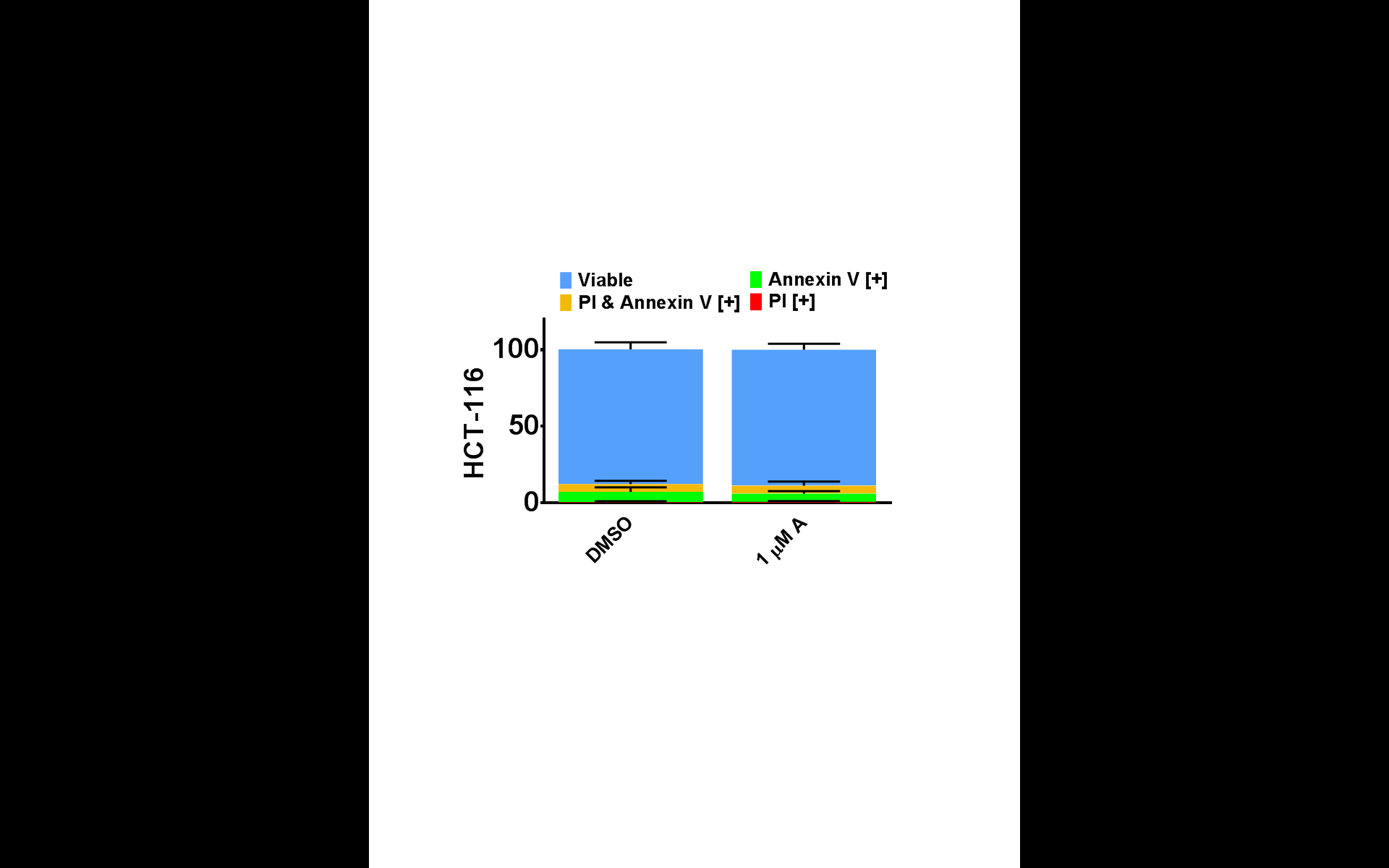


**Supplemental 9. No induction of Apoptosis Following Treatment at 6 Hours**. Cells were treated for 6 hours with the following drugs then stained with Annexin V and PI. Results were obtained using image-based cytometry with the Y-axis representative of percent of cells positive for Annexin V (green), PI (red), Annexin V and PI (yellow), or negative for both Annexin V and PI (blue). Results were quantified using the Tali Values are expressed as a mean ± SD from three independent experiments.


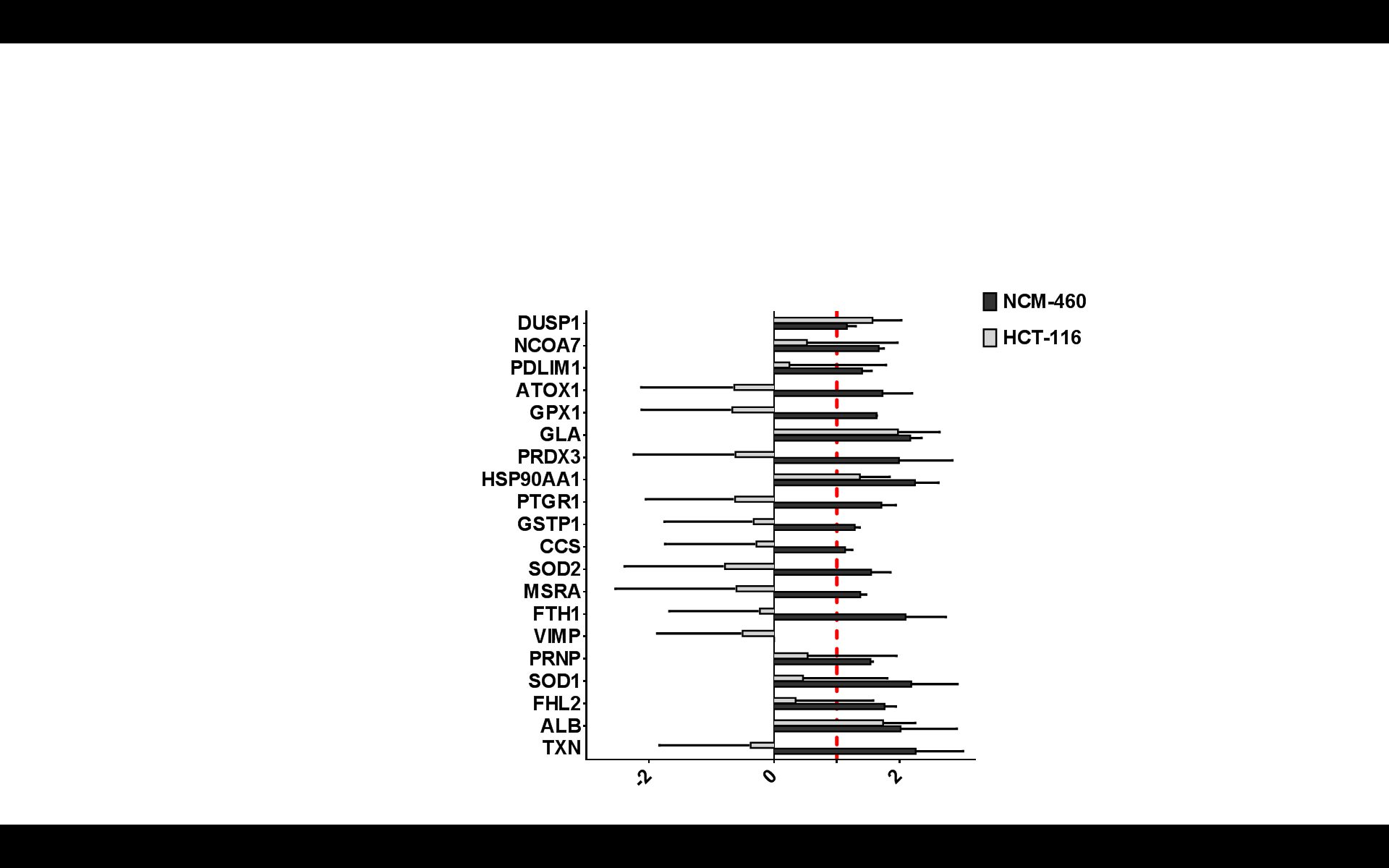


**Supplemental 10 – Statistically Non-Significant Changes in Gene Expression Following Treatment with Compound A.** – The cancerous cell line (HCT-116) and non-cancerous cell line (NCM-460) were treated with the vehicle control (DMSO) or 1 µM Compound A for 6 hours. RNA isolation and subsequent RT^2^ PCR analysis was carried out. Results are shown as a fold-change relative to control following treatment with Compound A of three independent trials. Statistical analysis was done using One-Way ANOVA compared to the DMSO (control) group. Results shown here are *p* > 0.05. Red dash line is set at a relative-fold of 1.


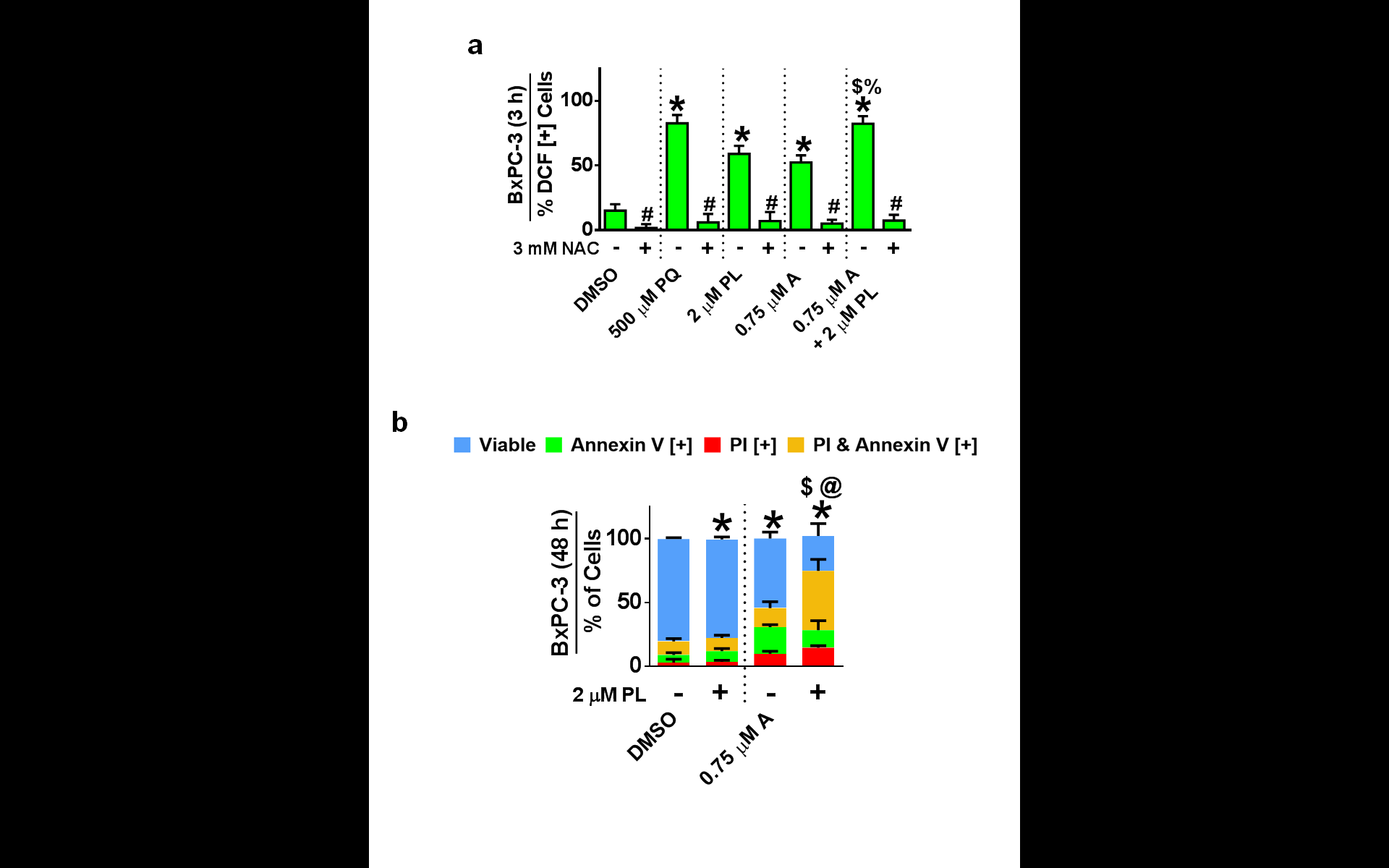


**Supplemental 11. Synergistic Apoptotic Effects with Piperlonguimine and Compound A in Combination Against Pancreatic Cancer Cell Line.** (**a**) BxPC-3 cells were treated with H_2_DCFDA then with Compound A either alone, in combination with NAC or PL alone, or both NAC and PL for 3 hours to monitor the production of ROS. Results were obtained using the image-based cytometry with the Y-axis representative of percent of cells positive for DCF. Values are expressed as a mean ± SD from three independent experiments. (**b**) BxPC-3 cells were treated with Compound A either alone, in combination with or without PL for 48 hours. Following treatment, cells were stained for Annexin V and PI. Results were obtained using image-based cytometry with the Y-axis representative of percent of cells positive for Annexin V (green), PI (red), Annexin V and PI (yellow), or negative for both Annexin V and PI (blue). Values are expressed as a mean ± SD from three independent experiments. Statistical calculations were performed using One-Way ANOVA multiple comparison for (**a**) or Two-Way ANOVA multiple comparison for (**b**). * p < 0.05 vs % viable or DCF [+] cells of control (DMSO); % p < 0.05 vs % viable or DCF [+] cells of group treated with PL , NAC, and Compound A; @ p< 0.05 vs % viable of group treated with Compound A; $ p < 0.05 vs % viable or DCF [+] cells of group treated with PL.


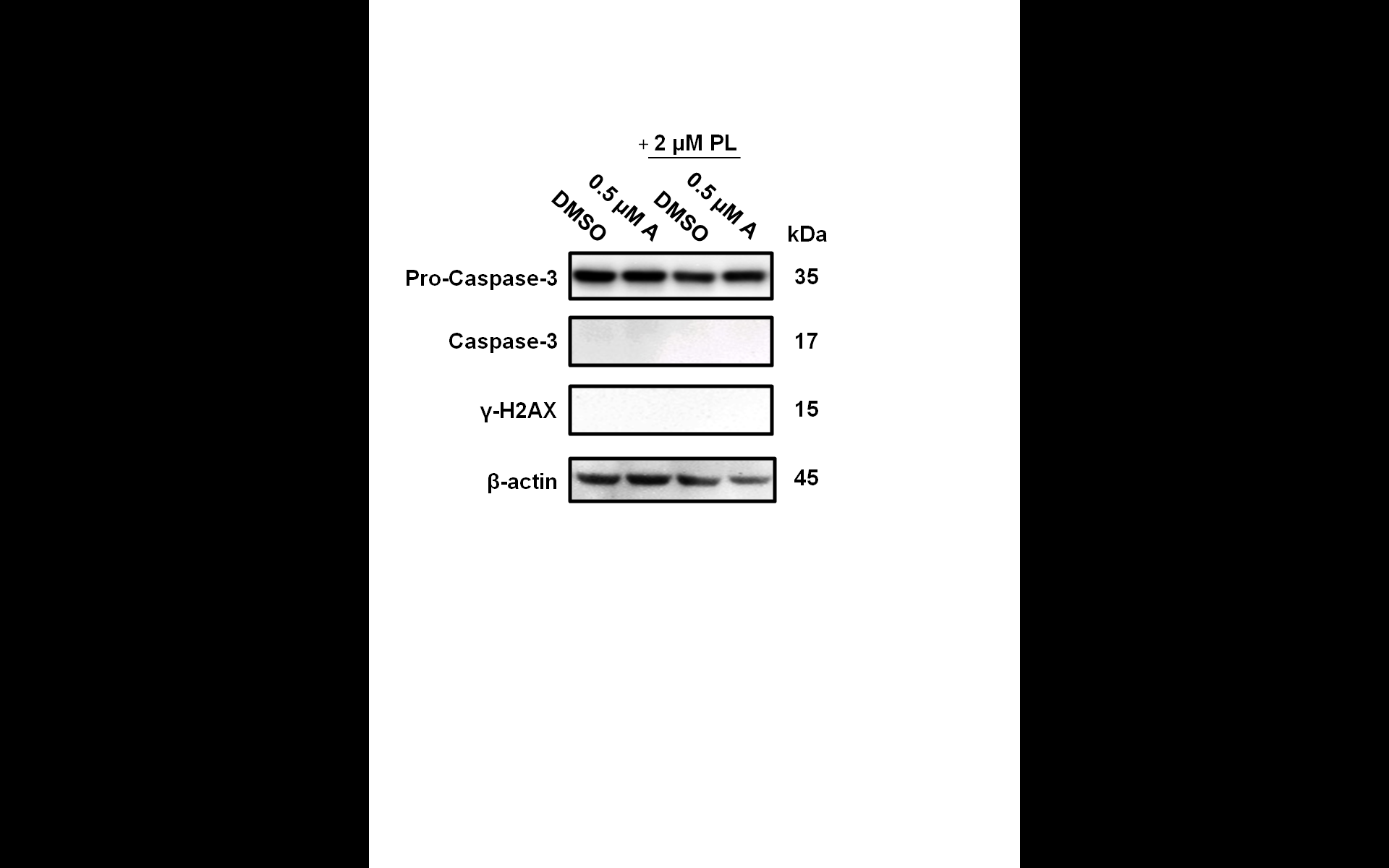


**Supplemental 12. Co-Treatment of Compound A and PL do not Induce Caspase-3 Cleavage or Double-Stranded DNA Damage in Normal Fibroblast Cells.** NHF cells were incubated with the following doses for 48 hours. Cells were lysed and protein samples were subjected to SDS-PAGE then transferred to a PVDF membrane. Bands were visualized with a chemiluminescence reagent. Images are representative of three independent experiments.


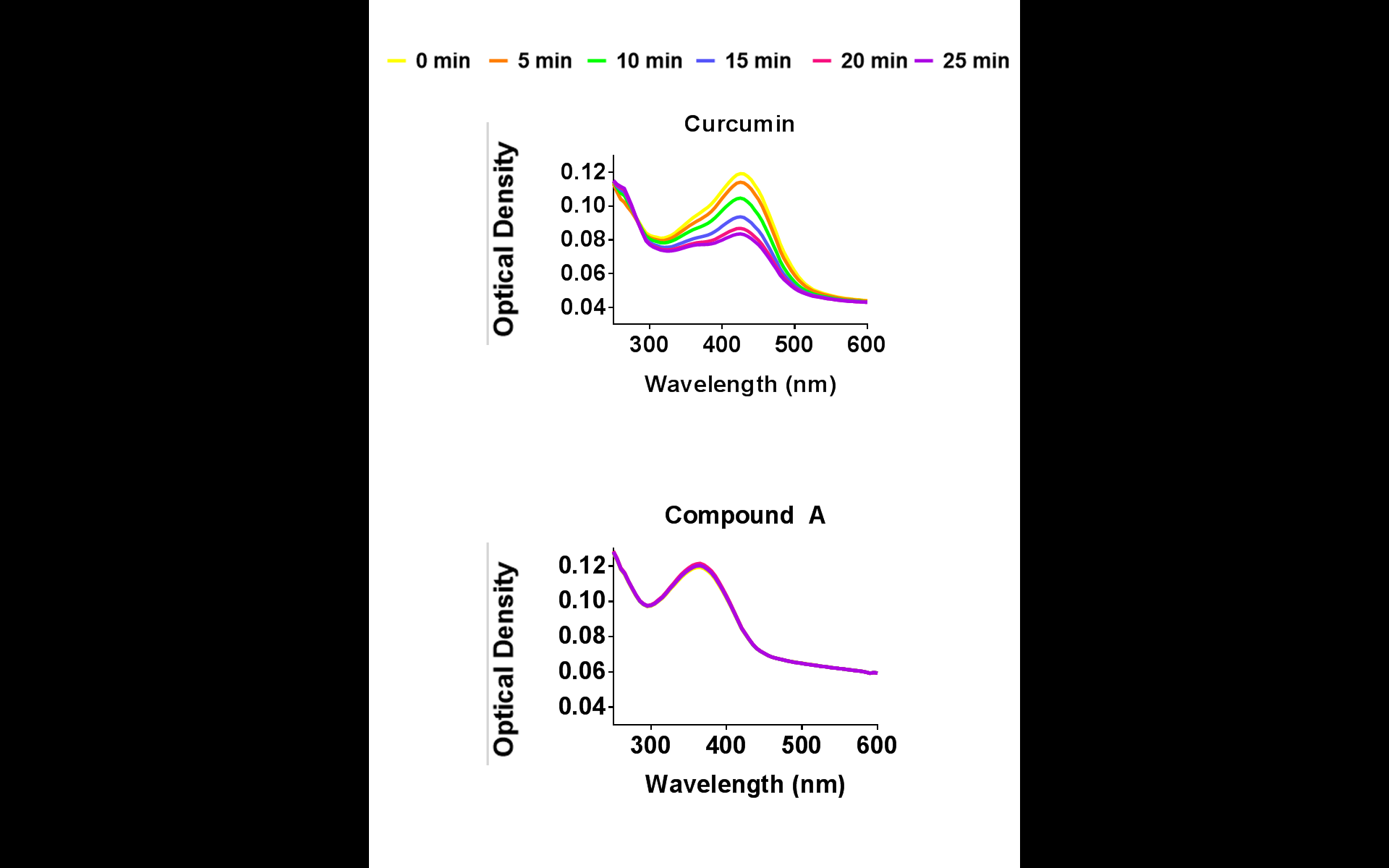


**Supplemental 13. Improved Chemical Stability of Compound A Compared to Natural Curcumin.** Curcumin and Compound A at 0.1 mM/L were dissolved in PBS in a cuvette and optical density values from 250 to 600 nm were determined using the Spectrum Max M5 at 5 minute intervals.


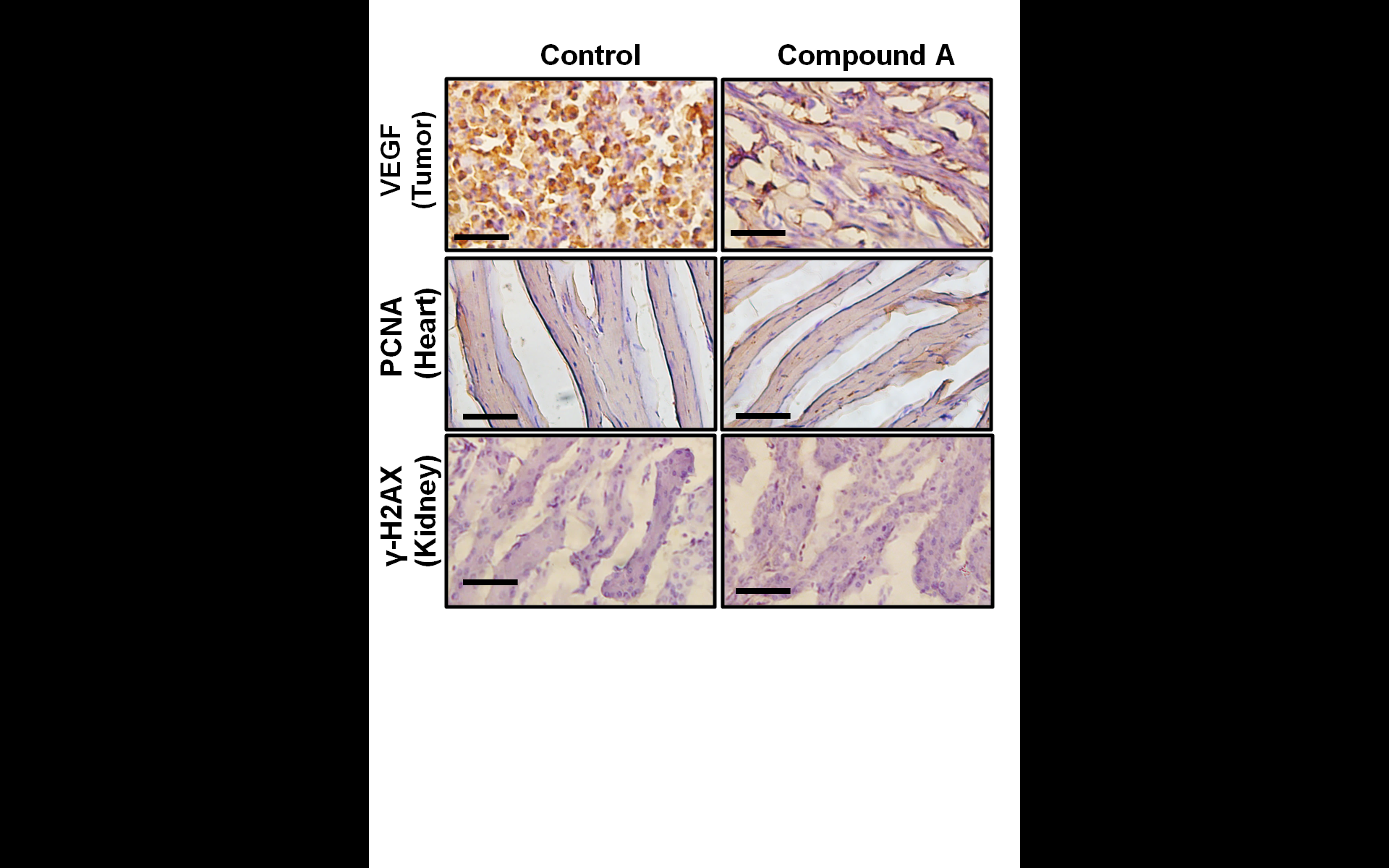


**Supplemental 14. Decrease in Tumor VEGF Quantity and No Significant Changes in Cellular Proliferation and DNA Damage Markers in Heart and Kidney Tissues.** Following the 5-week MDA-MB-231 study, mice were sacrificed and the tumors or organs were harvested, sectioned then subjected to immunohistochemistry for the following markers. Each section was subjected to the specified antibody followed by a biotinylated secondary antibody. Detection was done using a DAB Peroxidase HRP Substrate Kit (brown) followed by Hematoxylin counterstaining (purple). Images were obtained using inverted bright field microscopy. Scale bar is 50 microns. Results are representative of three independent sections of each tissue. Scale bar = 50 microns.


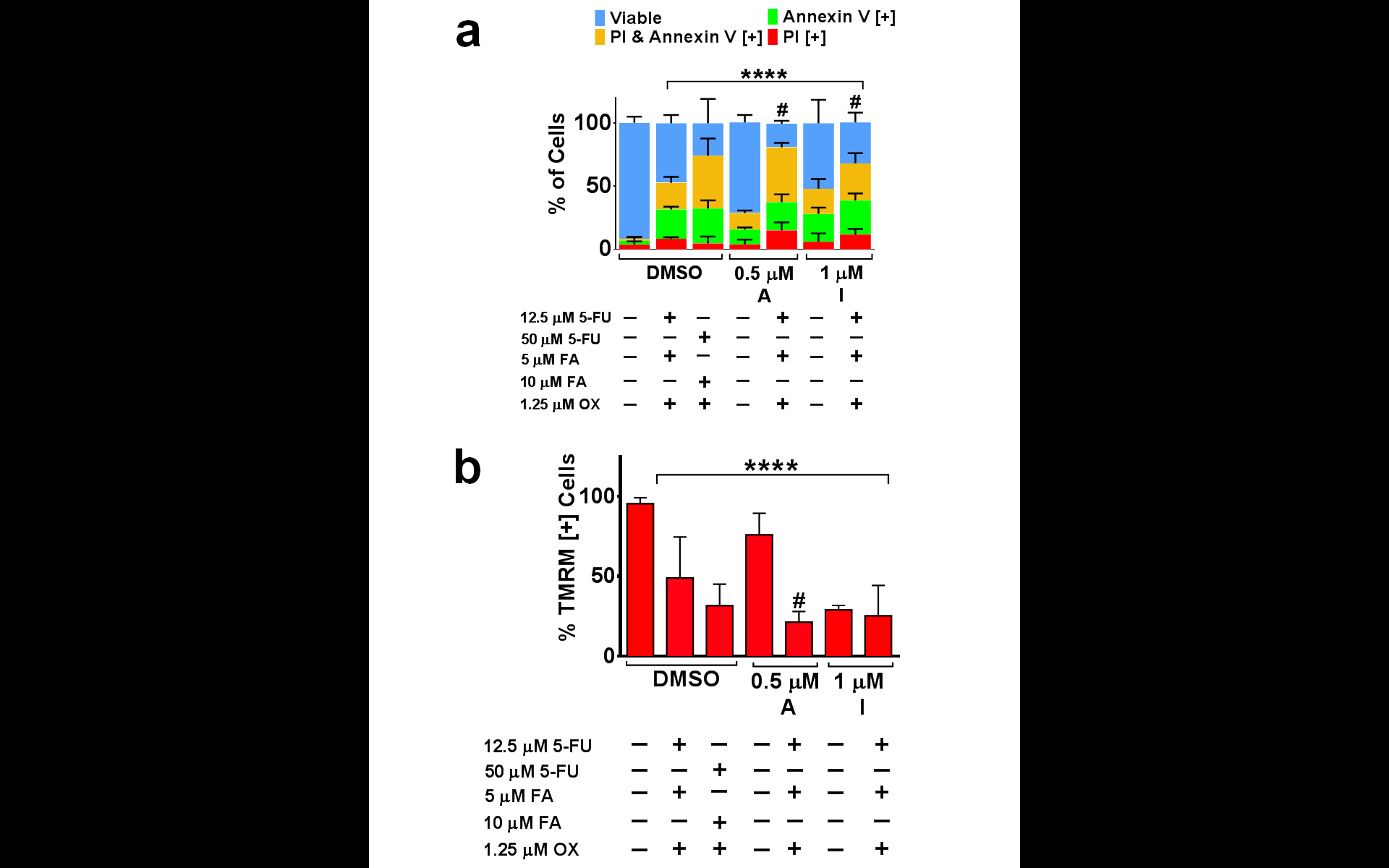


**Supplemental 15. Co-Treatment of Standard Chemotherapeutic Cocktail FOLFOX and Compound A or I Demonstrate an Additive Effect in Apoptotic Induction Towards Colon Cancer Cells.** Cells were treated for 72 hours with the following combination of drugs. (**a**) Annexin V and PI staining. Results were obtained using image-based cytometry with the Y-axis representative of percent of cells positive for Annexin V (green), PI (red), Annexin V and PI (yellow), or negative for both Annexin V and PI (blue). Results were quantified using the Tali Values are expressed as a mean ± SD from three independent experiments. (**b**) TMRM staining, which was obtained using image-based cytometry with the Y-axis representative of percent of cells positive for TMRM expressed as a mean ± SD from three independent experiments. Statistical calculations were performed using Two-Way ANOVA multiple comparison for (**a**) or One-Way ANOVA multiple comparison for (**b**). **** p < 0.001 vs. % viable or % positive TMRM cells for Control (DMSO); # p < 0.05 vs. % viable or % positive TMRM cells for Compound A or I treatment Alone.
